# Supplementary material for: SARS‐CoV‐2 spike protein enhances MAP4K3/GLK‐induced ACE2 stability in COVID‐19
Source: EMBO Mol Med. 2022 Jul 27;14(9):e15904. doi: 10.15252/emmm.202215904 (PMC9353388; doi:10.15252/emmm.202215904)
Supplement: Supplementary file 1 — Appendix [file EMMM-14-e15904-s005.pdf]

# Appendix Figures and Table

# SARS-CoV-2 spike protein stimulates MAP4K3/GLK-induced ACE2 stabilization in COVID-19

Huai-Chia Chuang, Chia-Hsin Hsueh, Pu-Ming Hsu, Rou-Huei Huang, Ching-Yi Tsai, Nai-Hsiang Chung, Yen-Hung Chow, Tse-Hua Tan

## Table of contents


| | |
|---|---|
| Appendix Figure S1 | Page 2 |
| Appendix Figure S2 | Page 3 |
| Appendix Figure S3 | Page 4 |
| Appendix Figure S4 | Page 5 |
| Appendix Figure S5 | Page 6 |
| Appendix Figure S6 | Page 7 |
| Appendix Figure S7 | Page 8 |
| Appendix Figure S8 | Page 9 |
| Appendix Table S1 | Page 10-12 |

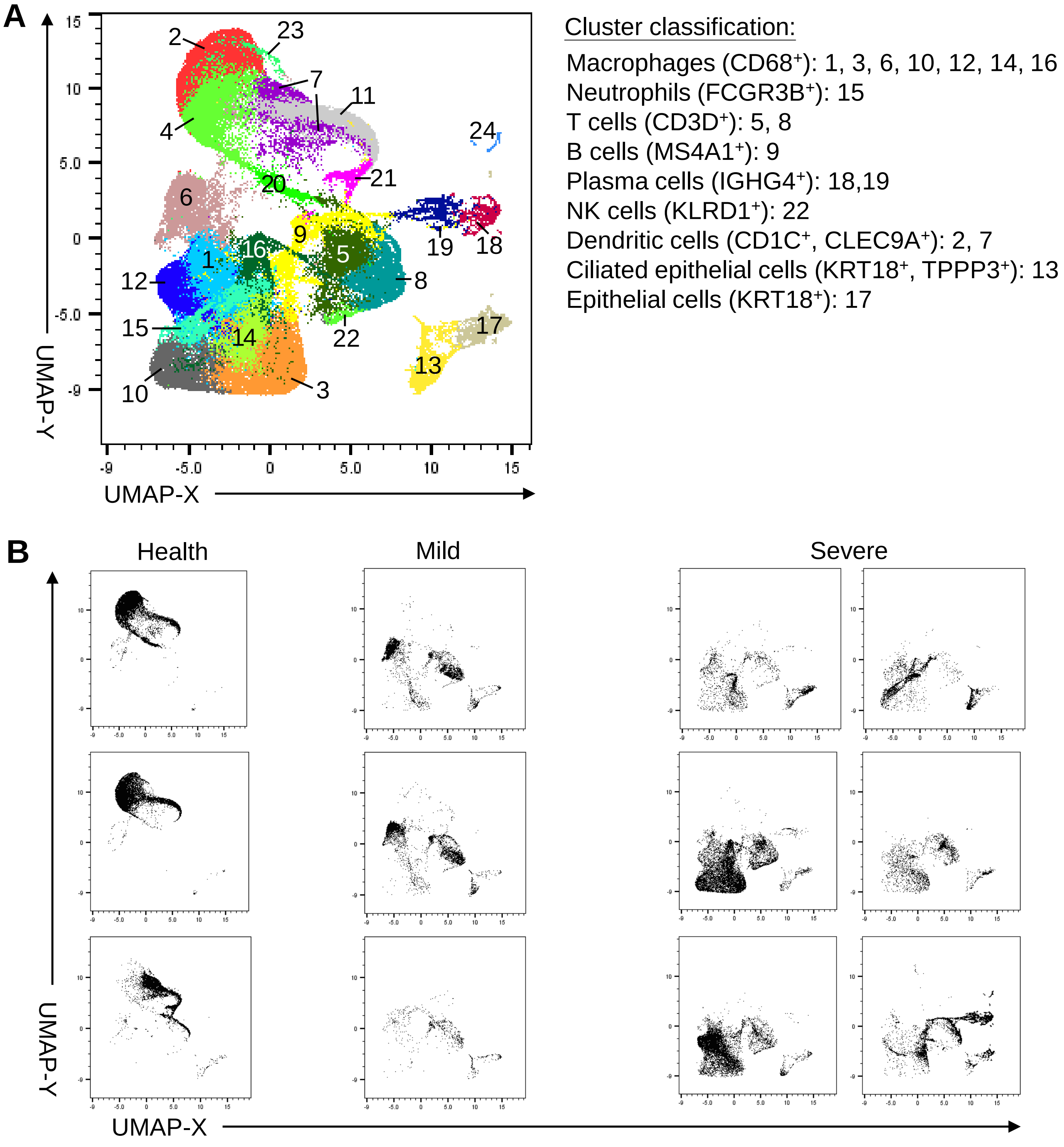


**Appendix Figure S1. Distribution and classification of individual cell types within human bronchoalveolar lavage fluid (BALF) cells.** (**A**) Distribution and classification of BALF cells from all 12 individuals of Cohort #1 (Reference #9). Data are shown in UMAP. (**B**) Individual distributions of BALF cells from 3 healthy controls (HC), 3 mild COVID-19 patients, and 6 severe COVID-19 patients. Cell populations were defined as described (Liao *et al*., 2020).

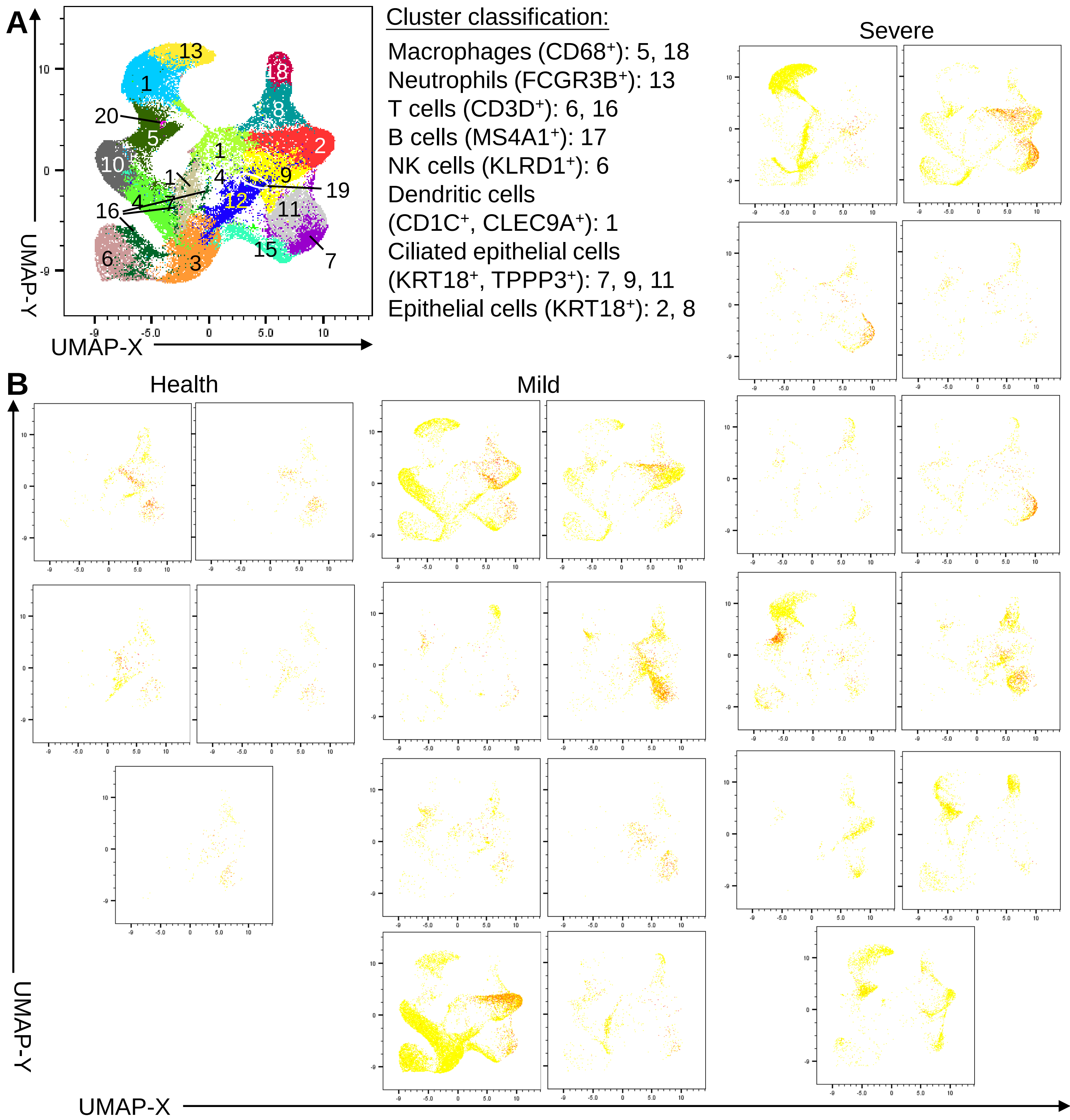


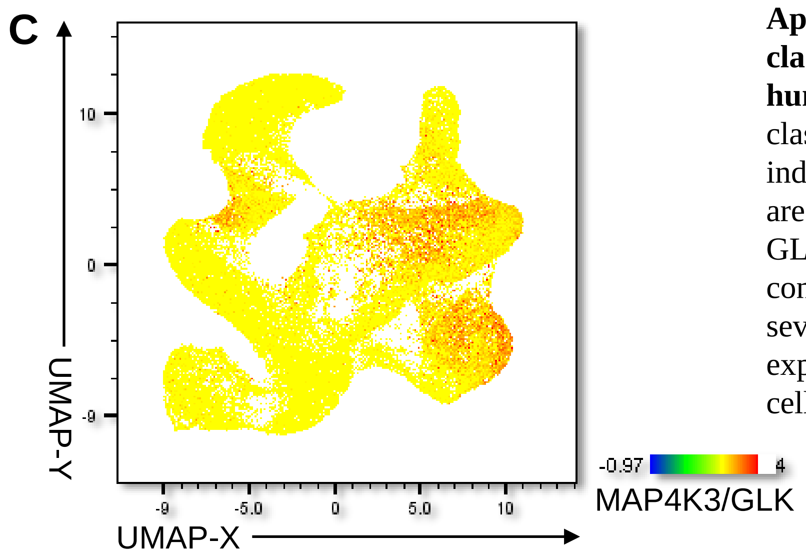


**Appendix Figure S2. Distribution and classification of individual cell types within human nasal swab cells.** (**A**) Distribution and classification of nasal swab cells from all 24 individuals of Cohort #2 (Chua *et al.*, 2020). Data are shown in UMAP. (**B**) Individual distributions of GLK-positive nasal swab cells from 5 healthy controls (HC), 8 mild COVID-19 patients, and 11 severe COVID-19 patients. (**C**) Single-cell gene expression of MAP4K3 (GLK) in all individual cells is shown in color scale.

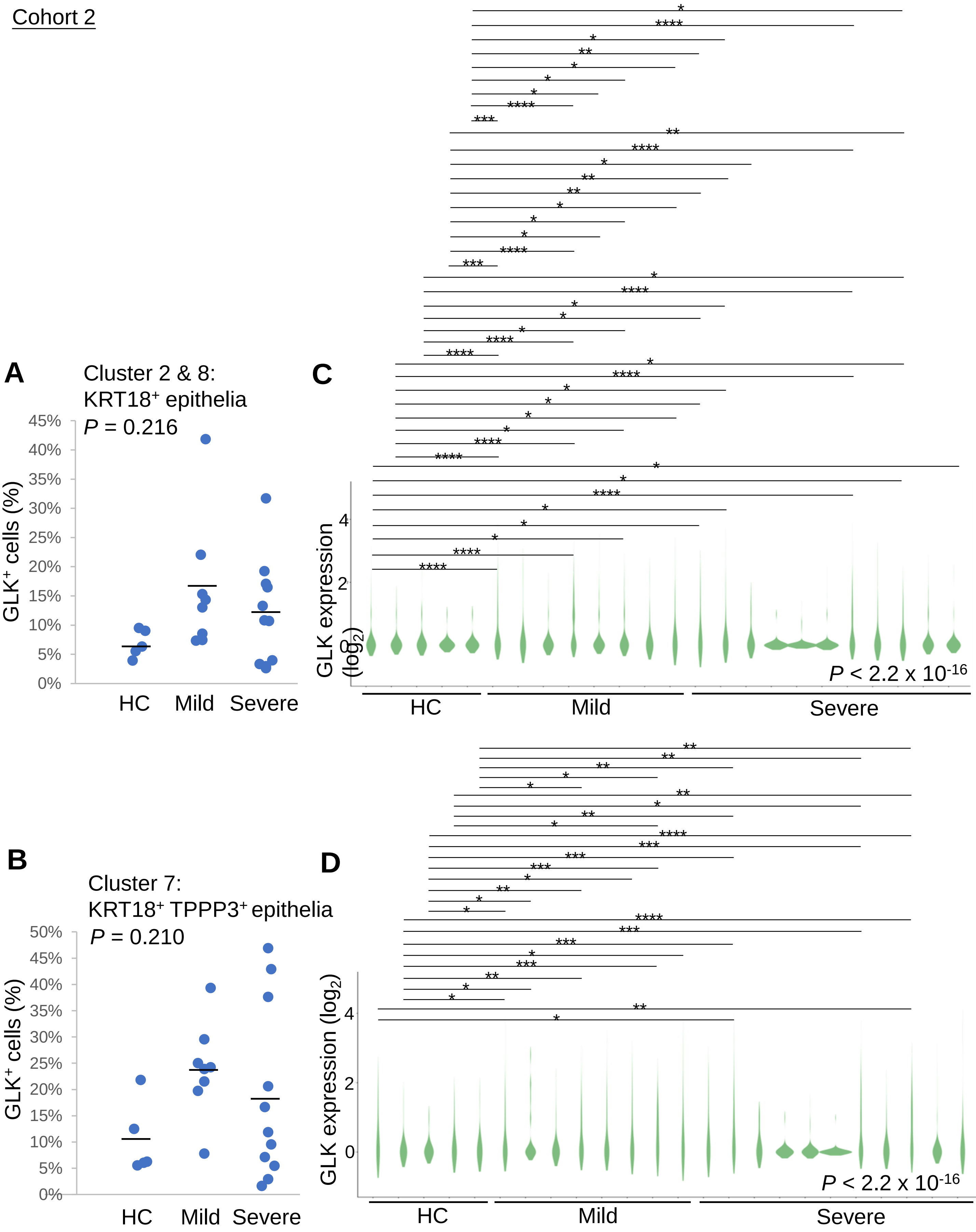


**Appendix Figure S3. GLK mRNA levels are increased in human nasal swab cells of COVID-19 patients.** (**A** and **B**) The percentages of MAP4K3/GLK-positive $KRT18^+$ epithelial (A) or $KRT18^+$ $TPPP3^+$ ciliated cells (B) in the nasopharynx from 5 healthy controls (HC), 8 mild COVID-19 patients, and 11 severe COVID-19 patients (Cohort #2). *, *P* value < 0.05 (ANOVA test). (**C** and **D**) GLK mRNA levels in $KRT18^+$ epithelial (C) or $KRT18^+$ $TPPP3^+$ ciliated epithelial cells (D) of Cohort #2. *, *P* value < 0.05; **, *P* value < 0.01; ***, *P* value < 0.001; ****, *P* value < 0.0001 (*Kruskal-Wallis* test).

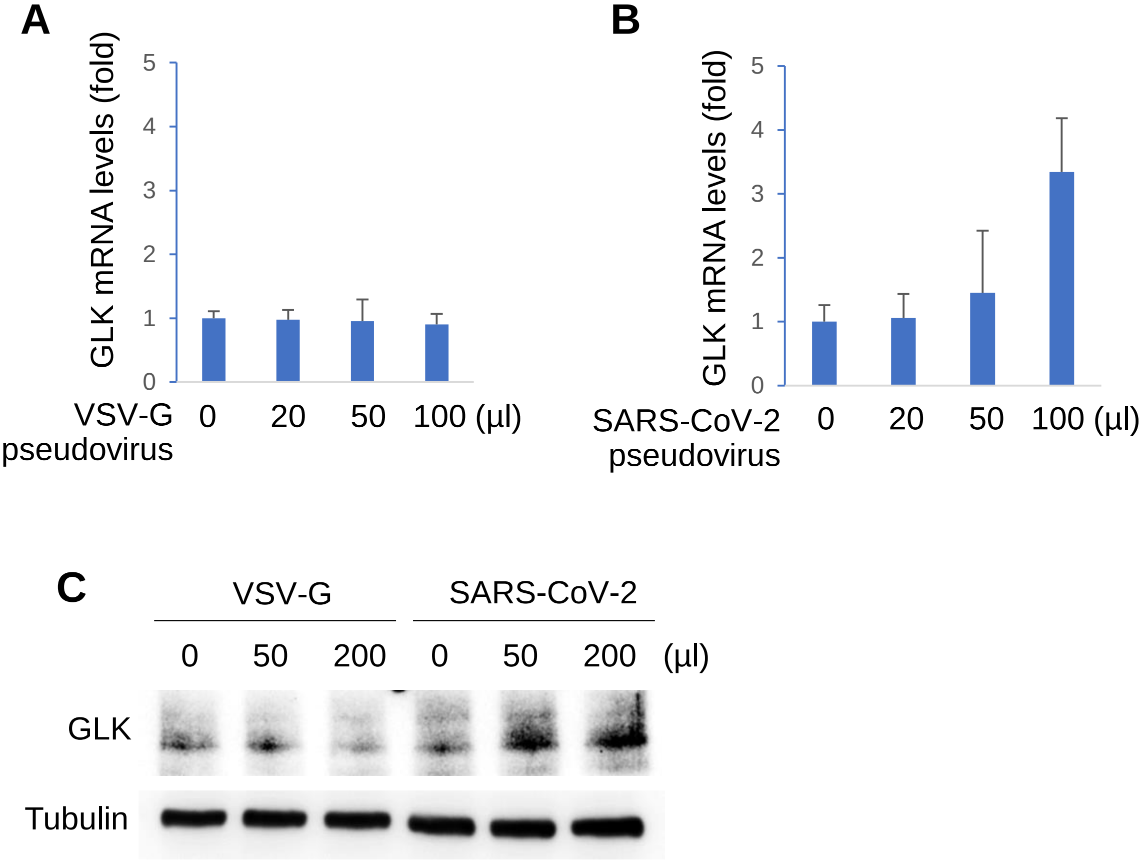


**Appendix Figure S4. GLK levels are increased by SARS-CoV-2 pseudovirus.** (**A** and **B**) Endogenous GLK levels were increased by SARS-CoV-2 pseudovirus infection. Real-time PCR analysis of mouse GLK mRNA levels in HCC827 lung epithelial cells either infected with VSV-G pseudotyped lentivirus (A) or SARS-CoV-2 pseudotyped lentivirus (B) for 24 h. The mRNA levels of GLK were normalized to GAPDH mRNA levels. Means ± SD are shown. Immunoblotting analysis of the endogenous GLK and tubulin proteins from the lysates of HCC827 lung epithelial cells either infected with VSV-G pseudovirus or SARS-CoV-2 pseudovirus for 24 h. Pseudovirus concentration: $1.2 \times 10^5$ copies/μl.

Cohort 3

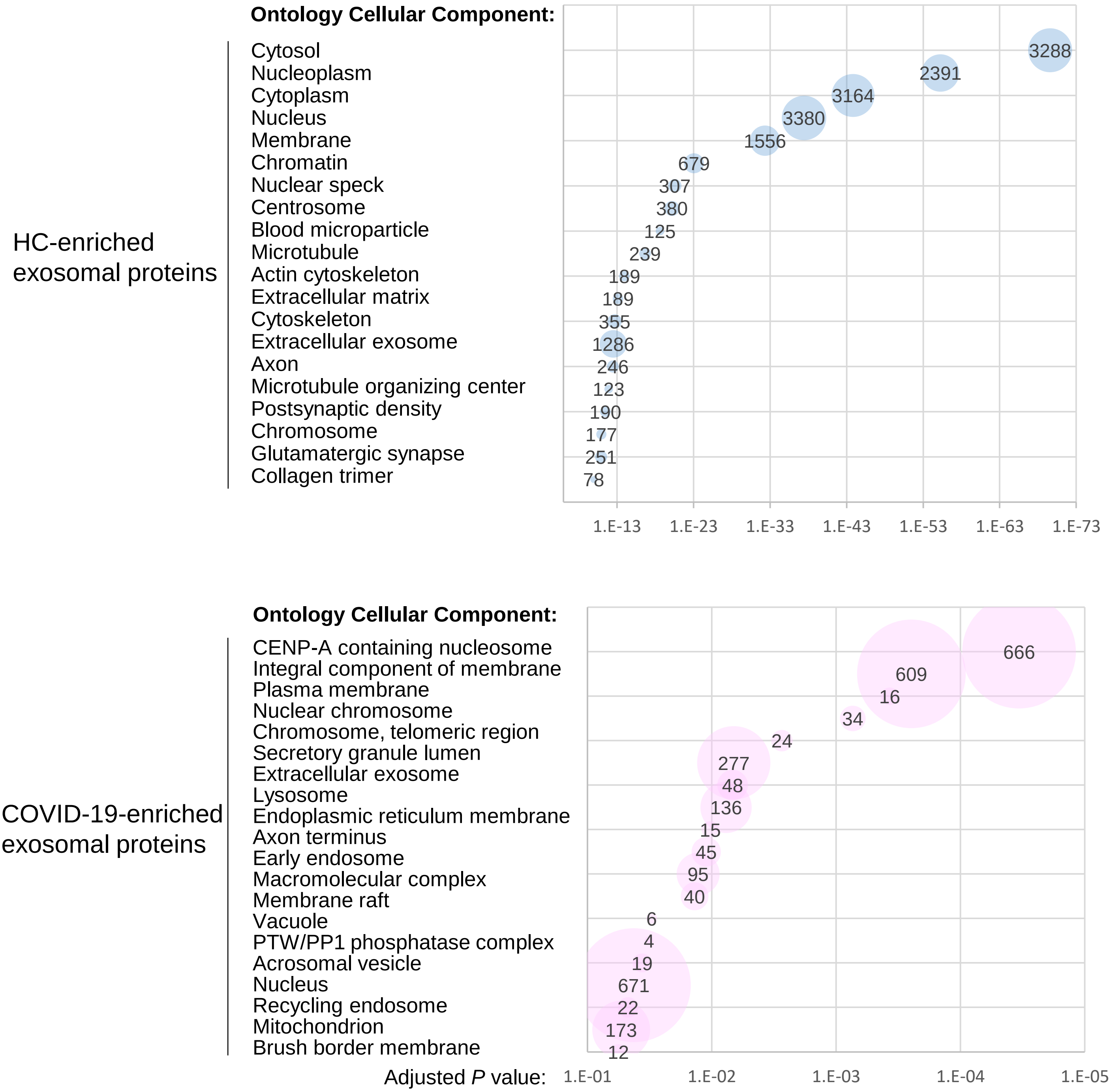


**Appendix Figure S5. COVID-19-enriched exosomal proteins mainly belong to membrane proteins by Ontology Cellular Component analysis.** (**A** and **B**) Ontology Cellular Component analysis of the healthy control (HC)-enriched exosomal proteins (A) and COVID-19 patient-enriched exosomal proteins (B). HC-enriched exosomal proteins were identified by mass spectrometry proteomics and then by excluding exosomal proteins identified from COVID-19 patients. COVID-19 patient-enriched exosomal proteins were identified by excluding exosomal proteins identified from HC. Cellular components belonging to different classifications are listed on the left side of the plot. Varied numbers of genes enriched in individual pathways are presented by different diameter sizes and numbers for individual dots. Adjusted *P* value is ranging from 0~1; less *P* value means greater intensiveness.
Source data are available online for this figure.

Cohort 3

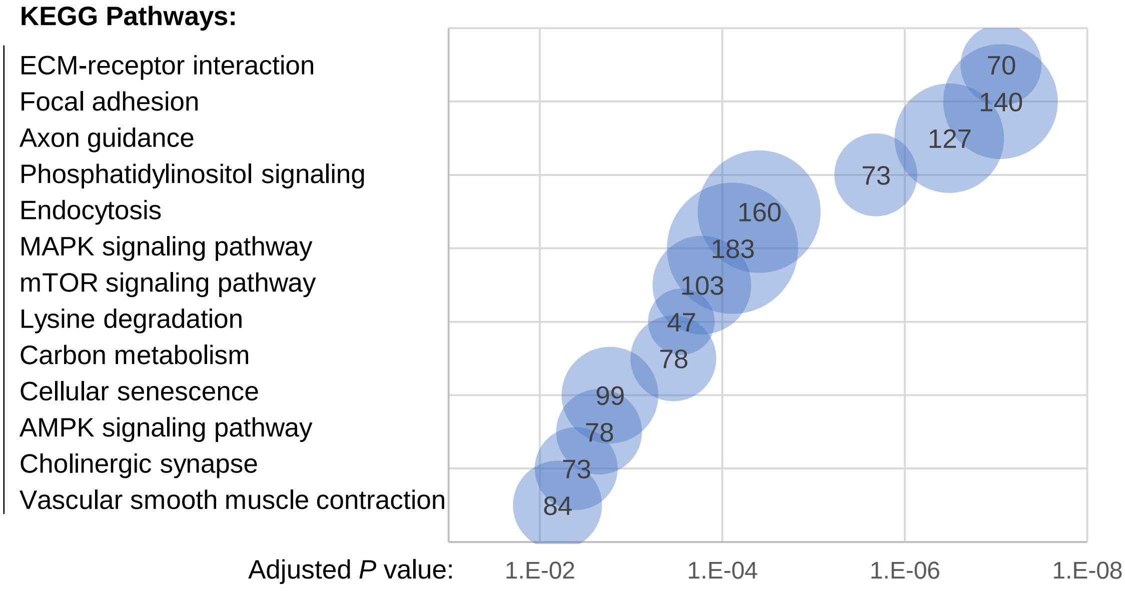


HC-enriched exosomal proteins

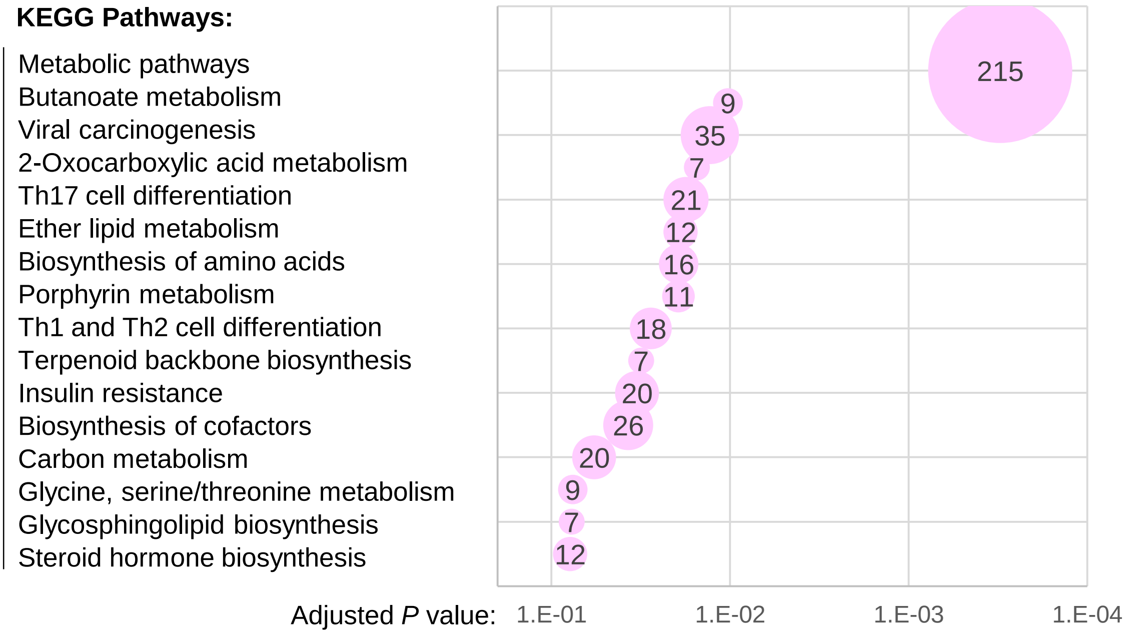


COVID-19-enriched exosomal proteins

**Appendix Figure S6. COVID-19 enriched exosomal proteins mainly belong to metabolic pathways by KEGG analysis.** (**A** and **B**) KEGG (Kyoto Encyclopedia of Genes and Genomes) enriched pathways of the healthy control (HC)-enriched exosomal proteins (A) and COVID-19 patient-enriched exosomal proteins (B). HC-enriched exosomal proteins were identified by mass spectrometry proteomics and then by excluding exosomal proteins identified from COVID-19 patients. COVID-19 patient-enriched exosomal proteins were identified by excluding exosomal proteins identified from HC. Pathways belonging to different classifications are listed on the left side of the plot. Varied numbers of genes enriched in individual pathways are presented by different diameter sizes and numbers for individual dots. Adjusted *P* value is ranging from 0~1; less *P* value means greater intensiveness.
Source data are available online for this figure.

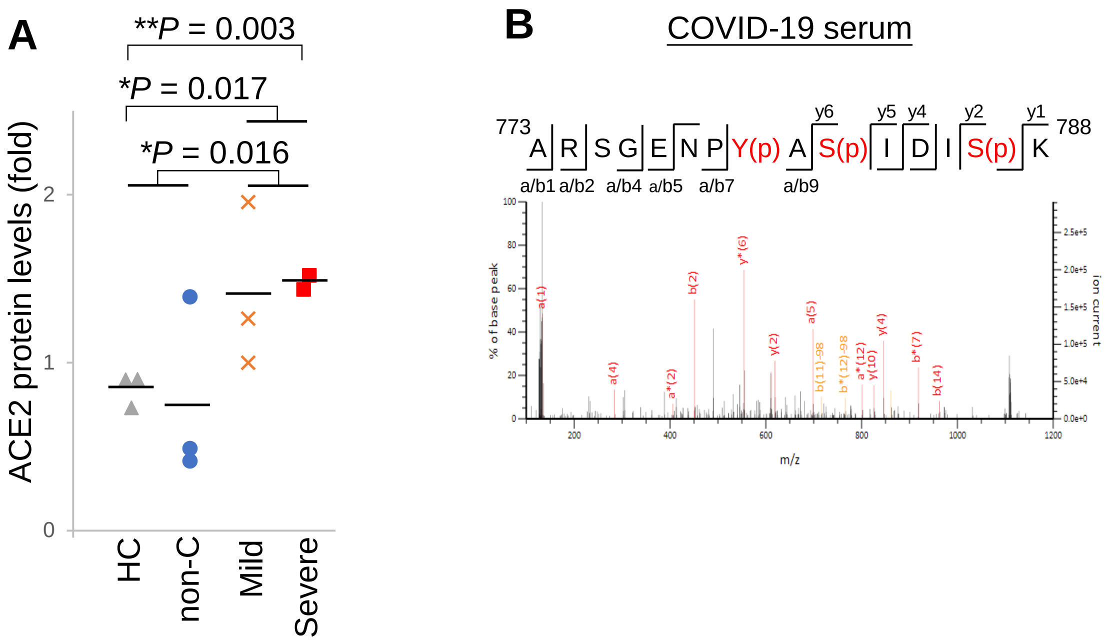


**Appendix Figure S7. Phosphorylated ACE2 proteins are detected in serum samples from COVID-19 patients.** (**A**) Relative levels of ACE2 proteins that were detected in serum samples from COVID-19 patients (batch 1 of Cohort #4) are calculated according to the TMTpro-labeled signals. HC, healthy controls; non-C, non-COVID-19 controls; mild, mild COVID-19 patients, severe, severe COVID-19 patients. (**B**) Mass spectrometry analysis of the ACE2 peptides containing Ser776 and Ser783 residues from COVID-19 patient sera. Phospho-Ser776, Ser783, and Tyr781 residues of ACE2 proteins detected in serum samples of COVID-19 patients (Cohort #4) are shown.

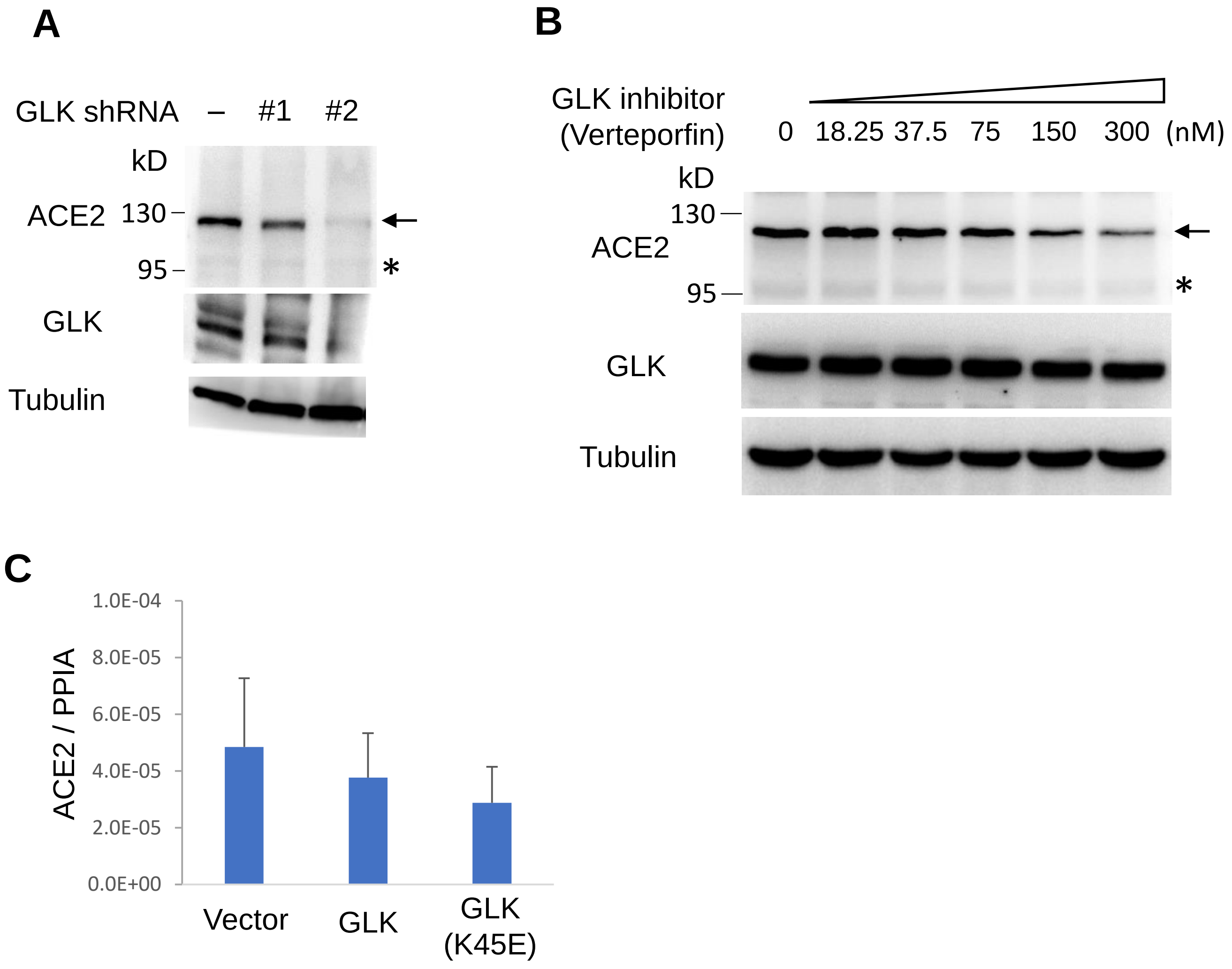


**Appendix Figure S8. Endogenous ACE2 protein levels are decreased by GLK shRNA knockdown or GLK inhibitor treatment.** (**A**) Immunoblotting analysis of the endogenous ACE2 and GLK proteins from the lysates of H661 lung epithelial cells transfected with GLK shRNA#1 or shRNA#2. Arrowhead denotes glycosylated ACE2 proteins; asterisk denotes unglycosylated ACE2 proteins. (**B**) Immunoblotting analysis of endogenous ACE2 and GLK proteins from the lysates of H661 lung epithelial cells treated with the GLK inhibitor verteporfin for 24 h. Arrowhead denotes glycosylated ACE2 proteins; asterisk denotes unglycosylated ACE2 proteins. (**C**) Human ACE2 mRNA levels in HCC827 lung epithelial cells transfected with vector, GLK, or GLK (K45E) kinase-dead mutant were analyzed by real-time PCR. The expression levels of ACE2 were normalized to peptidylprolyl isomerase A (PPIA) levels. Means ± SD are shown. Data shown are representative results of three independent experiments.

**Appendix Table S1. Human ACE2 peptides detected in sera of COVID-19 patients of Cohort #4**

| Start − End | Score | Human ACE2 Peptides |
|---|---|---|
| 2 − 26 | 15 | M.SSSSWLLLSLVAVTAAQSTIEEQAK.T + Phospho (ST); GlyGly (K) |
| 2 − 31 | 9 | M.SSSSWLLLSLVAVTAAQSTIEEQAKTFLDK.F + Phospho (ST); LeuArgGlyGly (K) |
| 2 − 31 | 17 | M.SSSSWLLLSLVAVTAAQSTIEEQAKTFLDK.F + 5 Phospho (ST); GlyGly (K) |
| 95 − 112 | 15 | K.LQLQALQQNGSSVLSEDK.S + Phospho (ST); GlyGly (K) |
| 95 − 112 | 17 | K.LQLQALQQNGSSVLSEDK.S |
| 113 − 131 | 15 | K.SKRLNTILNTMSTIYSTGK.V + 4 Phospho (ST) |
| 113 − 131 | 14 | K.SKRLNTILNTMSTIYSTGK.V + 5 Phospho (ST); Phospho (Y); GlyGly (K); LeuArgGlyGly (K) |
| 113 − 131 | 15 | K.SKRLNTILNTMSTIYSTGK.V + 6 Phospho (ST); Phospho (Y); GlyGly (K); LeuArgGlyGly (K) |
| 115 − 131 | 14 | K.RLNTILNTMSTIYSTGK.V + Phospho (ST); GlyGly (K) |
| 115 − 131 | 6 | K.RLNTILNTMSTIYSTGK.V + Phospho (ST); GlyGly (K) |
| 115 − 131 | 14 | K.RLNTILNTMSTIYSTGK.V + Phospho (ST); GlyGly (K) |
| 115 − 131 | 15 | K.RLNTILNTMSTIYSTGK.V + 2 Phospho (ST); GlyGly (K) |
| 115 − 131 | 15 | K.RLNTILNTMSTIYSTGK.V + Phospho (ST) |
| 116 − 131 | 13 | R.LNTILNTMSTIYSTGK.V + Phospho (ST); GlyGly (K) |
| 116 − 131 | 9 | R.LNTILNTMSTIYSTGK.V + Phospho (ST); GlyGly (K) |
| 116 − 131 | 14 | R.LNTILNTMSTIYSTGK.V + Phospho (ST); GlyGly (K) |
| 116 − 131 | 7 | R.LNTILNTMSTIYSTGK.V + Phospho (ST); GlyGly (K) |
| 116 − 131 | 19 | R.LNTILNTMSTIYSTGK.V + Phospho (ST); GlyGly (K) |
| 116 − 131 | 14 | R.LNTILNTMSTIYSTGK.V + Phospho (ST); GlyGly (K) |
| 116 − 131 | 11 | R.LNTILNTMSTIYSTGK.V + Phospho (ST); GlyGly (K) |
| 116 − 131 | 15 | R.LNTILNTMSTIYSTGK.V + 2 Phospho (ST); GlyGly (K) |
| 116 − 131 | 12 | R.LNTILNTMSTIYSTGK.V |
| 116 − 131 | 5 | R.LNTILNTMSTIYSTGK.V |
| 116 − 131 | 11 | R.LNTILNTMSTIYSTGK.V + Phospho (ST) |
| 170 − 187 | 12 | R.SEVGKQLRPLYEEYVVLK.N + Phospho (ST); 2 LeuArgGlyGly (K) |
| 205 − 219 | 9 | R.GDYEVNGVDGYDYSR.G + Phospho (ST) |
| 307 − 313 | 3 | R.IFKEAEK.F + 2 GlyGly (K) |
| 307 − 313 | 3 | R.IFKEAEK.F + 2 GlyGly (K) |
| 310 − 353 | 17 | K.EAEKFFVSVGLPNMTQGFWENSMLTDPGNVQKAVCHPTAWDLGK.G + Carbamidomethyl (C); 2 Phospho (ST); GlyGly (K); LeuArgGlyGly (K) |
| 310 − 353 | 14 | K.EAEKFFVSVGLPNMTQGFWENSMLTDPGNVQKAVCHPTAWDLGK.G + Carbamidomethyl (C); 2 Phospho (ST); GlyGly (K); LeuArgGlyGly (K) |

| | | |
|---|---|---|
| 354 – 363 | 7 | K.GDFRILMCTK.V + GlyGly (K) |
| 354 – 363 | 5 | K.GDFRILMCTK.V + GlyGly (K) |
| 354 – 363 | 5 | K.GDFRILMCTK.V + GlyGly (K) |
| 354 – 363 | 14 | K.GDFRILMCTK.V + GlyGly (K) |
| 420 – 441 | 17 | K.SIGLLSPDFQEDNETEINFLLK.Q + Phospho (ST); LeuArgGlyGly (K) |
| 420 – 458 | 15 | K.SIGLLSPDFQEDNETEINFLLKQALTIVGTLPFTYMLEK.W + 5 Phospho (ST) |
| 442 – 458 | 12 | K.QALTIVGTLPFTYMLEK.W + Phospho (ST); GlyGly (K) |
| 442 – 458 | 12 | K.QALTIVGTLPFTYMLEK.W + Phospho (ST) |
| 535 – 553 | 3 | K.HEGPLHKCDISNSTEAGQK.L + 2 Phospho (ST); 2 GlyGly (K) |
| 542 – 553 | 11 | K.CDISNSTEAGQK.L + Carbamidomethyl (C); GlyGly (K) |
| 542 – 553 | 6 | K.CDISNSTEAGQK.L + Phospho (ST); GlyGly (K) |
| 542 – 553 | 7 | K.CDISNSTEAGQK.L + Phospho (ST); GlyGly (K) |
| 542 – 553 | 15 | K.CDISNSTEAGQK.L + 3 Phospho (ST); GlyGly (K) |
| 542 – 553 | 16 | K.CDISNSTEAGQK.L + Carbamidomethyl (C); Phospho (ST) |
| 542 – 553 | 16 | K.CDISNSTEAGQK.L + Carbamidomethyl (C); Phospho (ST) |
| 560 – 577 | 18 | R.LGKSEPWTLALENVVGAK.N + GlyGly (K); LeuArgGlyGly (K) |
| 563 – 596 | 16 | K.SEPWTLALENVVGAKNMNVRPLLNYFEPLFTWLK.D + 2 Phospho (ST); 2 GlyGly (K) |
| 578 – 596 | 15 | K.NMNVRPLLNYFEPLFTWLK.D + Phospho (ST); LeuArgGlyGly (K) |
| 601 – 621 | 15 | K.NSFVGWSTDWSPYADQSIKVR.I + Phospho (ST); Phospho (Y); LeuArgGlyGly (K) |
| 601 – 621 | 13 | K.NSFVGWSTDWSPYADQSIKVR.I + 4 Phospho (ST) |
| 601 – 625 | 13 | K.NSFVGWSTDWSPYADQSIKVRISLK.S + Phospho (ST); Phospho (Y); 2 GlyGly (K) |
| 620 – 631 | 8 | K.VRISLKSALGDK.A + Phospho (ST); 2 GlyGly (K) |
| 620 – 631 | 11 | K.VRISLKSALGDK.A + 2 Phospho (ST); 2 GlyGly (K) |
| 620 – 631 | 13 | K.VRISLKSALGDK.A + Phospho (ST); GlyGly (K) |
| 620 – 631 | 3 | K.VRISLKSALGDK.A + Phospho (ST); GlyGly (K); LeuArgGlyGly (K) |
| 620 – 631 | 1 | K.VRISLKSALGDK.A + LeuArgGlyGly (K) |
| 622 – 631 | 10 | R.ISLKSALGDK.A + Phospho (ST); 2 GlyGly (K) |
| 622 – 631 | 12 | R.ISLKSALGDK.A + 2 Phospho (ST); 2 GlyGly (K) |
| 622 – 631 | 13 | R.ISLKSALGDK.A + 2 Phospho (ST); GlyGly (K); LeuArgGlyGly (K) |
| 626 – 644 | 15 | K.SALGDKAYEWNDNEMYLFR.S + GlyGly (K) |
| 645 – 657 | 8 | R.SSVAYAMRQYFLK.V + Phospho (ST); GlyGly (K) |
| 690 – 697 | 5 | K.NVSDIIPR.T + Phospho (ST) |
| 690 – 702 | 3 | K.NVSDIIPRTEVEK.A + GlyGly (K) |
| 690 – 702 | 12 | K.NVSDIIPRTEVEK.A + GlyGly (K) |
| 709 – 716 | 2 | R.SRINDAFR.L + Phospho (ST) |
| 709 – 716 | 3 | R.SRINDAFR.L + Phospho (ST) |

| | | |
|---|---|---|
| 709 – 716 | 6 | R.SRINDAFR.L + Phospho (ST) |
| 709 – 716 | 4 | R.SRINDAFR.L + Phospho (ST) |
| 772 – 788 | 16 | K.NKARSGENPYASIDISK.G + 2 GlyGly (K) |
| 772 – 788 | 13 | K.NKARSGENPYASIDISK.G + 2 GlyGly (K) |
| 772 – 788 | 3 | K.NKARSGENPYASIDISK.G + GlyGly (K) |
| 774 – 788 | 22 | K.ARSGENPYASIDISK.G + GlyGly (K) |
| 774 – 788 | 16 | K.ARSGENPYASIDISK.G + GlyGly (K) |
| 774 – 788 | 15 | K.ARSGENPYASIDISK.G + GlyGly (K) |
| 774 – 788 | 13 | K.ARSGENPYASIDISK.G + GlyGly (K) |
| 774 – 788 | 10 | K.ARSGENPYASIDISK.G + GlyGly (K) |
| 774 – 788 | 12 | K.ARSGENPYASIDISK.G + GlyGly (K) |
| 774 – 788 | 10 | K.ARSGENPYASIDISK.G + GlyGly (K) |
| 774 – 788 | 7 | K.ARSGENPYASIDISK.G + Phospho (ST); GlyGly (K) |
| 774 – 788 | 15 | K.ARSGENPYASIDISK.G + Phospho (Y); GlyGly (K) |
| 774 – 788 | 7 | K.ARSGENPYASIDISK.G + Phospho (Y); GlyGly (K) |
| 774 – 788 | 12 | K.ARSGENPYASIDISK.G + Phospho (ST); GlyGly (K) |
| 774 – 788 | 17 | K.ARSGENPYASIDISK.G + Phospho (ST); GlyGly (K) |
| 774 – 788 | 9 | K.ARSGENPYASIDISK.G + Phospho (ST); GlyGly (K) |
| 774 – 788 | 14 | K.ARSGENPYASIDISK.G + Phospho (ST); GlyGly (K) |
| 774 – 788 | 12 | K.ARSGENPYASIDISK.G + 2 Phospho (ST); GlyGly (K) |
| 774 – 788 | 19 | K.ARSGENPYASIDISK.G + Phospho (ST); Phospho (Y); GlyGly (K) |
| 774 – 788 | 15 | K.ARSGENPYASIDISK.G + 2 Phospho (ST); GlyGly (K) |
| 774 – 788 | 21 | K.ARSGENPYASIDISK.G + 2 Phospho (ST); GlyGly (K) |
| 774 – 788 | 17 | K.ARSGENPYASIDISK.G + Phospho (ST); LeuArgGlyGly (K) |
| 774 – 788 | 12 | K.ARSGENPYASIDISK.G + Phospho (ST); LeuArgGlyGly (K) |
| 776 – 788 | 22 | R.SGENPYASIDISK.G + GlyGly (K) |
| 776 – 788 | 12 | R.SGENPYASIDISK.G + GlyGly (K) |
| 776 – 788 | 7 | R.SGENPYASIDISK.G + Phospho (ST); GlyGly (K) |
| 776 – 788 | 9 | R.SGENPYASIDISK.G + 2 Phospho (ST); GlyGly (K) |
| 776 – 788 | 15 | R.SGENPYASIDISK.G + Phospho (ST); Phospho (Y) |
| 776 – 788 | 11 | R.SGENPYASIDISK.G + 3 Phospho (ST); Phospho (Y); LeuArgGlyGly (K) |
