# Supplementary material for: SARS‐CoV‐2 spike protein enhances MAP4K3/GLK‐induced ACE2 stability in COVID‐19
Source: EMBO Mol Med. 2022 Jul 27;14(9):e15904. doi: 10.15252/emmm.202215904 (PMC9353388; doi:10.15252/emmm.202215904)
Supplement: Supplementary file 2 — Expanded View Figures PDF [file EMMM-14-e15904-s010.pdf]

Expanded View Figures

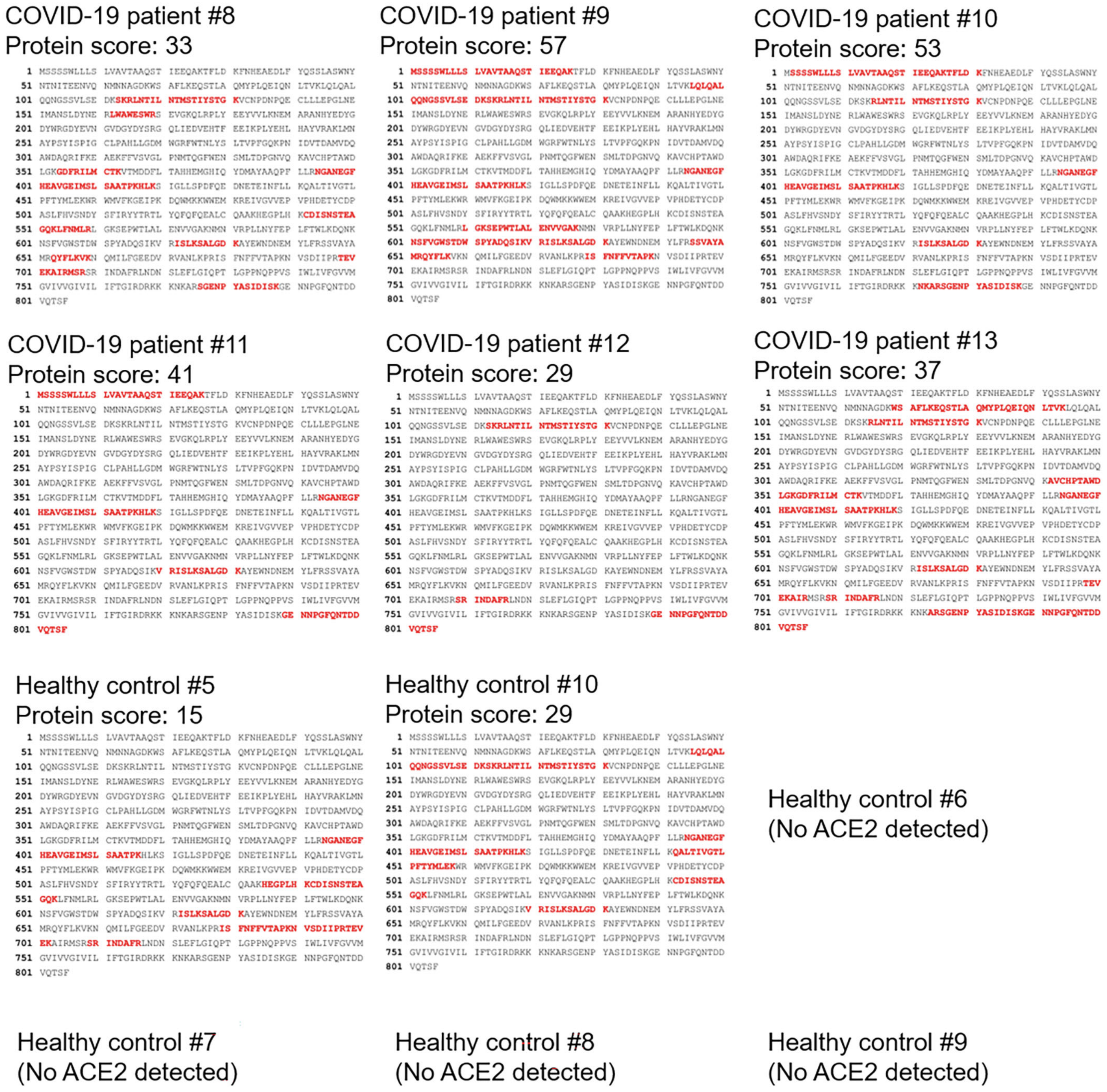

Figure EV1. ACE2 proteins are detected in the serum exosomes of all six COVID-19 patients.

The identified peptide sequences (red color) of endogenous ACE2 by mass spectrometry analyses using exosomes isolated from the sera of six COVID-19 patients and six healthy controls (Cohort #3). All six COVID-19 patients but only two of six healthy controls showed exosomal ACE2 proteins in the serum exosomes. Exosomes were sequentially isolated using ExoQuick kits and then ExoQuick ULTRA columns. Exosomes were further purified by immunoprecipitation using a combination of anti-CD9, anti-CD63, and anti-CD81 magnetic beads. The protein score is the sum of the highest ions score of MS/MS search for each distinct peptide.

© 2022 The Authors

A UBR4 protein sequence

```
1 MATSGGEEAA AAAPAPGTPA TGADTTPGWE VAVRPLLSAS YSAFEMKELP
51 QLVASVIESE SEILHHEKQY EPFYSSFVAL STHYITTVCS LIPRNQLQSV
101 AAACKVLIEF SLLRLENPDE ACAVSQKHLI LLIKGLCTGC SRLDRTEIIT
151 FTAMMKSAKL PQTVKTLSDV EDQKELASPV SPELRQKEVQ MNFLNQLTSV
201 FNPRTVASQP ISTQTLVEGE NDEQSSTDQA SAIKTKNVFI AQNVASLQEL
251 GGSEKLLRVC LNLPYFLRYI NRFQDAVLAN SFFIMPATVA DATAVRNGFH
301 SLVIDVTMAL DTLSLPVLEP LNPSRLQDVT VLSLSCLYAG VSVATCMAIL
351 HVGSAQQVRT GSTSSKEDDY ESDAATIVQK CLEIYDMIGQ AISSSRRAGG
401 EHYQNFQLLG AWCLLNSLFL ILNLSPTALA DKGKEKDPLA ALRVRDILSR
451 TKEGVGSPKL GPGKGHQGFG VLSVILANHA IKLLTSLFQD LQVEALHKGW
501 ETDGPPAALS IMAQSTSIQR IQRLIDSVPL MNLLLTLLST SYRKACVLQR
551 QRKGSMSSDA SASTDSNTYY EDDFSSTEED SSQDDDSEPI LGQWFEETIS
601 PSKEKAAPPP PPPPPLESS PRVKSPSKQA PGEKGNILAS RKDPELFLGL
651 ASNILNFITS SMLNSRNNFI RNYLSVSLSE HHMATLASII KEVDKDGLKG
701 SSDEEFAAAL YHFNHSLVTS DLQSPNLQNT LLQQLGVAPF SEGPWPLYIH
751 PQSLSVLSRL LLIWQHKASA QGDPDVPECL KVWDRFLSTM KQNALQGVVP
801 SETEDLNVEH LQMLLLIFHN FTETGRRAIL SLFVQIIQEL SVNMDAQMRF
851 VPLILARLLL IFDYLLHQYS KAPVYLFEQV QHNLLSPPFG WASGSQDSNS
901 RRATTPLYHG FKEVEENWSK HFSSDAVPHP RFYCVLSPEA SEDDLNRLDS
951 VACDVLFSKL VKYDELYAAL TALLAAGSQL DTVRRKENKN VTALEACALQ
1001 YYFLILWRIL GILPPSKTYI NQLSMNSPEM SECDILHTLR WSSRLRISSY
1051 VNWIKDHLIK QGMKAEHASS LLELASTTKC SSVKYDVEIV EEYFARQISS
1101 FCSIDCTTIL QLHEIPSLQS IYTLDAAISK VQVSLDEHFS KMAAETDPHK
1151 SSEITKNLLP ATLQLIDTYA SFTRAYLLQN FNEEGTTEKP SKEKLQGFAA
1201 VLAIGSSRCK ANTLGPTLVQ NLPSSVQTVC ESWNNINTNE FPNIGSWRNA
1251 FANDTIPSES YISAVQAAHL GTLCSQSLPL AASLKHTLLS LVRLTGDLIV
1301 WSDEMNPPQV IRTLLPLLLE SSTESVAEIS SNSLERILGP AESDEFLARV
1351 YEKLITGCYN ILANHADPNS GLDESILEEC LQYLEKQLES SQARKAMEEF
1401 FSDSGELVQI MMATANENLS AKFCNRVLKF FTKLFQLTEK SPNPSLLHLC
1451 GSLAQLACVE PVRLQAWLTR MTTSPPKDSD QLDVIQENRQ LLQLLTTYIV
1501 RENSQVGEGV CAVLLGTLTP MATEMLANGD GTGFPELMVV MATLASAGQG
1551 AGHLQLHNAA VDWLSRCKKY LSQKNVVEKL NANVMHGKHV MILECTCHIM
1601 SYLADVTNAL SQSNGQGPSH LSVDGEERAI EVDSDWVEEL AVEEEDSQAE
1651 DSDEDSLCNK LCTFTITQKE FMNQHWYHCH TCKMVDGVGV CTVCAKVCHK
1701 DHEISYAKYG SFFCDCGAKE DGSCLALVKR TPSSGMSSTM KESAFQSEPR
1751 ISESLVRHAS TSSPADKAKV TISDGKVADE EKPKKSSLCR TVEGCREELQ
1801 NQANFSFAPL VLDMLNFLMD AIQTNFQQAS AVGSSSRAQQ ALSELHTVEK
1851 AVEMTDQLMV PTLGSQEGAF ENVRMNYSGD QGQTIRQLIS AHVLRRVAMC
1901 VLSSPHGRRQ HLAVSHEKGK ITVLQLSALL KQADSSKRKL TLTRLASAPV
1951 PFTVLSLTGN PCKEDYLAVC GLKDCHVLTF SSSGSVSDHL VLHPQLATGN
2001 FIIKAVWLPG SQTELAIVTA DFVKIYDLCV DALSPTFYFL LPSSKIRDVT
2051 FLFNEEGKNI IVIMSSAGYI YTQLMEEASS AQQGPFYVTN VLEINHEDLK
2101 DSNSQVAGGG VSVYYSHVLQ MLFFSYCQGK SFAATISRTT LEVLQLFPIN
2151 IKSSNGGSKT SPALCQWSEV MNHPGLVCCV QQTTGVPLVV MVKPDTFLIQ
2201 EIKTLPAKAK IQDMVAIRHT ACNEQQRTTM ILLCEDGSLR IYMANVENTS
2251 YWLQPSLQPS SVISIMKPVR KRKTATITTR TSSQVTFPID FFEHNQQLTD
2301 VEFGGNDLLQ VYNAQQIKHR LNSTGMYVAN TKPGGFTIEI SNNNSTMVMT
2351 GMRIQIGTQA IERAPSYIEI FGRTMQLNLS RSRWFDFPFT REEALQADKK
2401 LNLFIGASVD PAGVTMIDAV KIYGKTKEQF GWPDEPPEEF PSASVSNICP
2451 SNLNQSNGTG DSDSAAPTTT SGTVLERLVV SSLEALESCF AVGPIIEKER
2501 NKNAAQELAT LLLSLPAPAS VQQQSKSLLA SLHTSRSAYH SHKDQALLSK
2551 AVQCLNTSSK EGKDLDPEVF QRLVITARSI AIMRPNNLVH FTESKLPQME
2601 TEGMDEGKEP QKQLEGDCCS FITQLVNHFW KLHASKPKNA FLAPACLPGL
2651 THIEATVNAL VDIIHGYCTC ELDCINTASK IYMQMLLCPD PAVSFSCKQA
2701 LIRVLRPRNK RRHVTLPSSP RSNTPMGDKD DDDDDDADEK MQSSGIPNGG
2751 HIRQESQEQS EVDHGDFEMV SESMVLETAE NVNNGNPSPL EALLAGAEGF
2801 PPMLDIPPDA DDETMVELAI ALSLQQDQQG SSSSALGLQS LGLSGQAPSS
2851 SSLDAGTLSD TTASAPASDD EGSTAATDGS TLRTSPADHG GSVGSESGGS
2901 AVDSVAGEHS VSGRSSAYGD ATAEGHPAGP GSVSSSTGAI STTTGHQEGD
2951 GSEGEGEGET EGDVHTSNRL HMVRLMLLER LLQTLPQLRN VGGVRAIPYM
3001 QVILMLTTDL DGEDEKDKGA LDNLLSQLIA ELGMDKKDVS KKNERSALNE
3051 VHLVVMRLLS VFMSRTKSGS KSSICESSSL ISSATAAALL SSGAVDYCLH
3101 VLKSLLEYWK SQQNDEEPVA TSQLLKPHTT SSPPDMSPFF LRQYVKGHAA
3151 DVFEAYTQLL TEMVLRLPYQ IKKITDTNSR IPPPVFDHSW FYFLSEYLMI
3201 QQTPFVRRQV RKLLLFICGS KEKYRQLRDL HTLDSHVRGI KKLLEEQGIF
3251 LRASVVTASS GSALQYDTLI SLMEHLKACA EIAAQRTINW QKFCIKDDSV
3301 LYFLLQVSFL VDEGVSPVLL QLLSCALCGS KVLAALAASS GSSSASSSSA
3351 PVAASSGQAT TQSKSSTKKS KKEEKEKEKD GETSGSQEDQ LCTALVNQLN
3401 KFADKETLIQ FLRCFLLESN SSSVRWQAHC LTLHIYRNSS KSQQELLLDL
3451 MWSIWPELPA YGRKAAQFVD LLGYFSLKTP QTEKKLKEYS QKAVEILRTQ
3501 NHILTNHPNS NIYNTLSGLV EFDGYYLESD PCLVCNNPEV PFCYIKLSSI
3551 KVDTRYTTTQ QVVKLIGSHT ISKVTVKIGD LKRTKMVRTI NLYYNNRTVQ
3601 AIVELKNKPA RWHKAKKVQL TPGQTEVKID LPLPIVASNL MIEFADFYEN
3651 YQASTETLQC PRCSASVPAN PGVCGNCGEN VYQCHKCRSI NYDEKDPFLC
3701 NACGFCKYAR FDFMLYAKPC CAVDPIENEE DRKKAVSNIN TLLDKADRVY
3751 HQLMGHRPQL ENLLCKVNEA APEKPQDDSG TAGGISSTSA SVNRYILQLA
3801 QEYCGDCKNS FDELSKIIQK VFASRKELLE YDLQQREAAT KSSRTSVQPT
3851 FTASQYRALS VLGCGHTSST KCYGCASAVT EHCITLLRAL ATNPALRHIL
3901 VSQGLIRELF DYNLRRGAAA MREEVRQLMC LLTRDNPEAT QQMNDLIIGK
3951 VSTALKGHWA NPDLASSLQY EMLLLTDSIS KEDSCWELRL RCALSLFLMA
4001 VNIKTPVVVE NITLMCLRIL QKLIKPPAPT SKKNKDVPVE ALTTVKPYCN
4051 EIHAQAQLWL KRDPKASYDA WKKCLPIRGI DGNGKAPSKS ELRHLYLTEK
4101 YVWRWKQFLS RRGKRTSPLD LKLGHNNWLR QVLFTPATQA ARQAACTIVE
4151 ALATIPSRKQ QVLDLLTSYL DELSIAGECA AEYLALYQKL ITSAHWKVYL
4201 AARGVLPYVG NLITKEIARL LALEEATLST DLQQGYALKS LTGLLSSFVE
4251 VESIKRHFKS RLVGTVLNGY LCLRKLVVQR TKLIDETQDM LLEMLEDMTT
4301 GTESETKAFM AVCIETAKRY NLDDYRTPVF IFERLCSIIY PEENEVTEFF
4351 VTLEKDPQQE DFLQGRMPGN PYSSNEPGIG PLMRDIKNKI CQDCDLVALL
4401 EDDSGMELLV NNKIISLDLP VAEVYKKVWC TTNEGEPMRI VYRMRGLLGD
4451 ATEEFIESLD STTDEEEDEE EVYKMAGVMA QCGGLECMLN RLAGIRDFKQ
4501 GRHLLTVLLK LFSYCVKVKV NRQQLVKLEM NTLNVMLGTL NLALVAEQES
4551 KDSGGAAVAE QVLSIMEIIL DESNAEPLSE DKGNLLLTGD KDQLVMLLDQ
4601 INSTFVRSNP SVLQGLLRII PYLSFGEVEK MQILVERFKP YCNFDKYDED
4651 HSGDDKVFLD CFCKIAAGIK NNSNGHQLKD LILQKGITQN ALDYMKKHIP
4701 SAKNLDADIW KKFLSRPALP FILRLLRGLA IQHPGTQVLI GTDSIPNLHK
4751 LEQVSSDEGI GTLAENLLEA LREHPDVNKK IDAARRETRA EKKRMAMAMR
4801 QKALGTLGMT TNEKGQVVTK TALLKQMEEL IEEPGLTCCI CREGYKFQPT
4851 KVLGIYTFTK RVALEEMENK PRKQQGYSTV SHFNIVHYDC HLAAVRLARG
4901 REEWESAALQ NANTKCNGLL PVWGPHVPES AFATCLARHN TYLQECTGQR
4951 EPTYQLNIHD IKLLFLRFAM EQSFSADTGG GGRESNIHLI PYIIHTVLYV
5001 LNTTRATSRE EKNLQGFLEQ PKEKWVESAF EVDGPYYFTV LALHILPPEQ
5051 WRATRVEILR RLLVTSQARA VAPGGATRLT DKAVKDYSAY RSSLLFWALV
5101 DLIYNMFKKV PTSNTEGGWS CSLAEYIRHN DMPIYEAADK ALKTFQEEFM
5151 PVETFSEFLD VAGLLSEITD PESFLKDLLN SVP
```

B

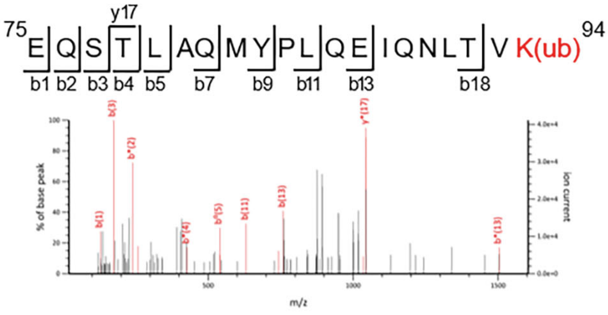

Figure EV2. UBR4 is identified as an ACE2-interacting protein by mass spectrometry.

A The identified peptide sequences (red color) of endogenous UBR4 by mass spectrometry analyses using ACE2 immunocomplexes. ACE2-overexpressing HEK293T cells were treated with the proteasome inhibitor MG132 (25 μM) for 2 h.

B Besides Lys26, Lys112, and Lys114 residues, Lys94 residue was also identified as an ACE2 ubiquitination site. Mass spectrometry analysis of the ACE2 peptide containing the ubiquitination residue Lys94 from ACE2-transfected HEK293T cells treated with MG132.

 © 2022 The Authors

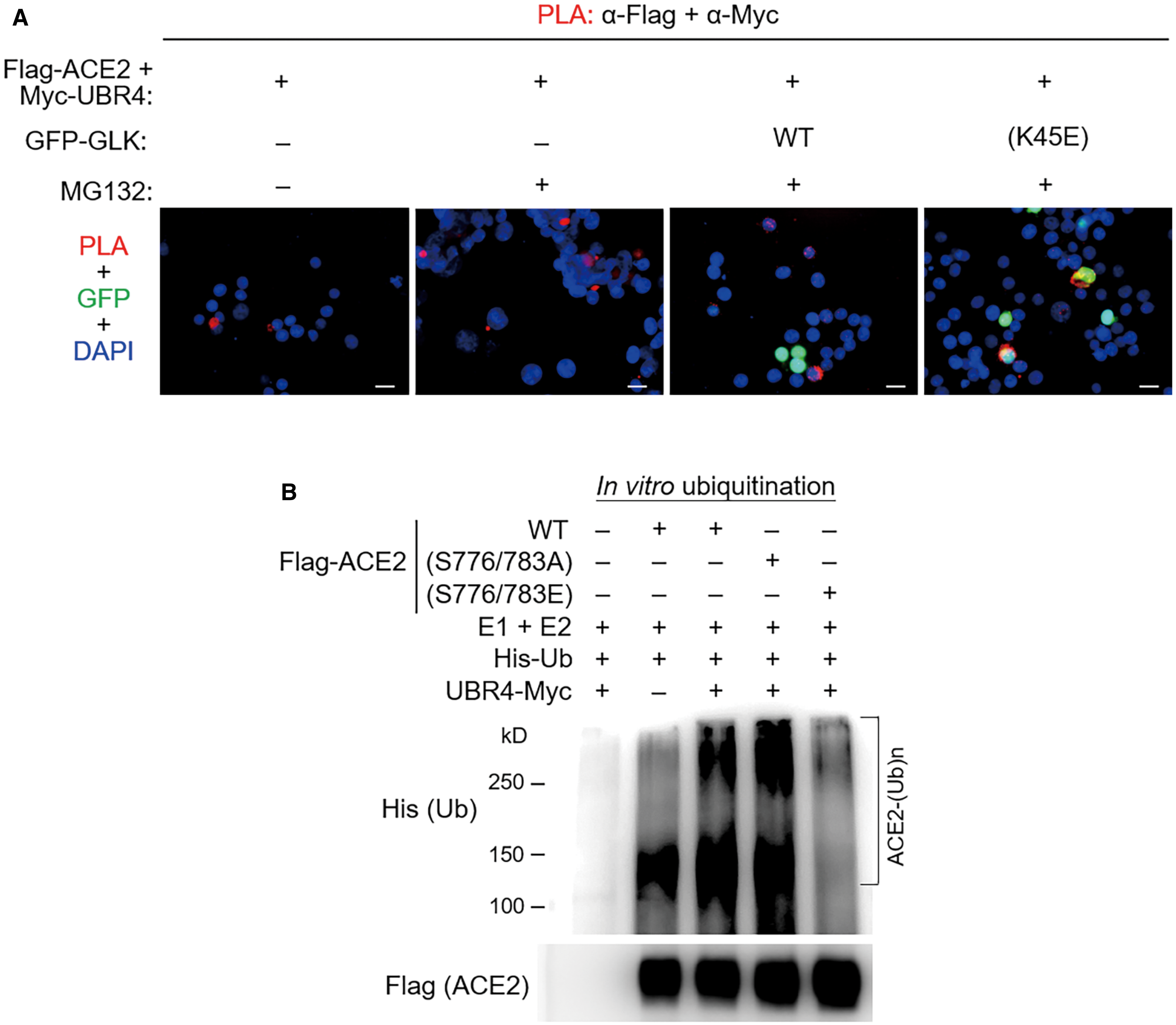

Figure EV3. GLK-induced ACE2 phosphorylation blocks the ACE2-UBR4 interaction and subsequent ACE2 ubiquitination.

A *In situ* PLA assays of the interaction between Flag-tagged ACE2 and Myc-tagged UBR4 proteins in HEK293T cells. Cells were co-transfected with Flag-ACE2 and Myc-UBR2 plus either GFP-GLK (green) or GFP-GLK (K45E) kinase-dead mutant (green) plasmids. Nuclei were stained with DAPI. Red dots represent direct interaction signals. Yellow color represents the co-existence of PLA signal (red) and GLK (K45E) mutant protein (green). Original magnification, ×200. Scale bars, 20 µm.

B *In vitro* ubiquitination assays using Myc-tagged UBR4 and Flag-tagged ACE2 proteins with E1 ubiquitin-activating enzyme, E2 ubiquitin-conjugating enzyme, and His-tagged ubiquitin. ACE2 ubiquitination and ACE2 were detected by immunoblotting with anti-His and anti-Flag antibodies, respectively. Data shown are representative results of three independent experiments.

© 2022 The Authors

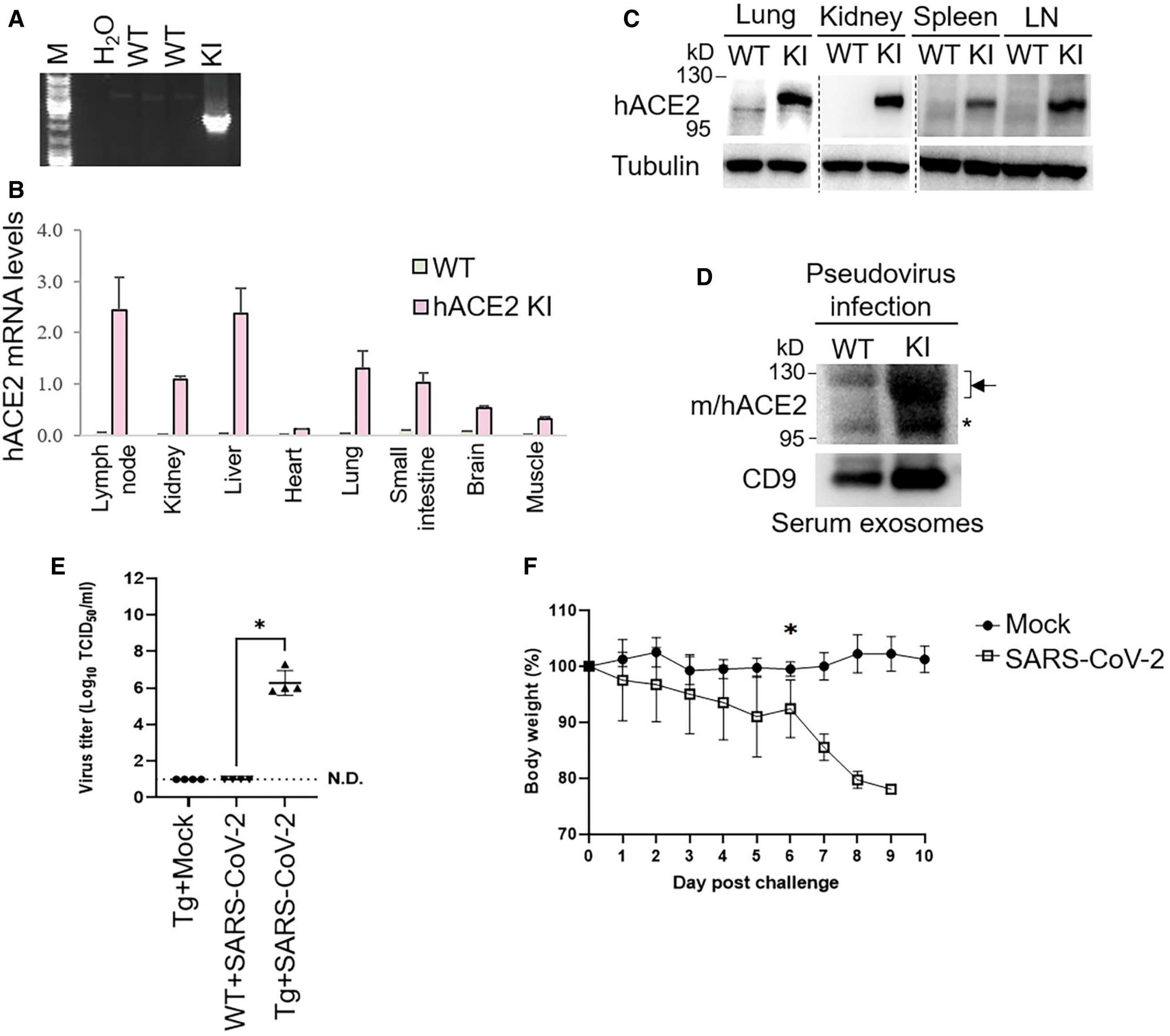

Figure EV4. Infection of hACE2 knockin mice and EF1α-hACE2 transgenic mice with SARS-CoV-2 pseudovirus or live SARS-CoV-2.

A PCR analyses of hACE2 knockin allele in the genomic DNA from mouse tails. The PCR product of the 2,379-bp band indicates the hACE2 knockin allele.

B Real-time PCR analyses of knockin human ACE2 (hACE2) mRNA levels in the lymph nodes, kidneys, liver, heart, lungs, small intestine, brain, and muscle from wild-type or hACE2 KI mice. The human ACE2 mRNA levels were normalized to mouse GAPDH mRNA levels. $n = 2$ (technical replicates) per group.

C Immunoblotting analyses of GLK, tubulin, and human ACE2 proteins in the lungs, kidneys, spleen, and lymph nodes of wild-type and hACE2 KI mice.

D Immunoblotting of ACE2, GLK, and CD9 proteins in the exosomes isolated from the sera of the SARS-CoV-2 pseudovirus-infected mice. Exosomes were isolated sequentially using ExoQuick kits and then ExoQuick ULTRA columns. Arrowheads denote glycosylated ACE2 proteins; asterisk denotes unglycosylated ACE2 proteins.

E, F EF1α-hACE2 Tg and wild-type mice were infected intranasally with 2×10^5 pfu of live SARS-CoV-2. $n = 4$ (biological replicates) per group. EF1α-hACE Tg mice were treated intranasally with PBS (Mock) as controls. On day 3 postinfection, the mice were sacrificed, and the lung tissues were homogenized for the $TCID_{50}$ assay (E). The survival rate of EF1α-hACE2 Tg mice challenged with live SARS-CoV2 was monitored (F). WT, wild-type mice; KI, hACE2 knockin mice; Tg, EF1α-hACE2 transgenic mice. The number of infected mice was less than 3 from day 7 due to deaths; thus, the statistical analysis of panel F was only performed on data up to day 6 postinfection.

Data information: In (B), data are presented as means ± SD. In (E), data are presented as means ± SEM. *P-value < 0.05 (Kruskal–Wallis test). In (F), data are presented as means ± SEM. *P-value < 0.05 (Mann–Whitney test).

© 2022 The Authors

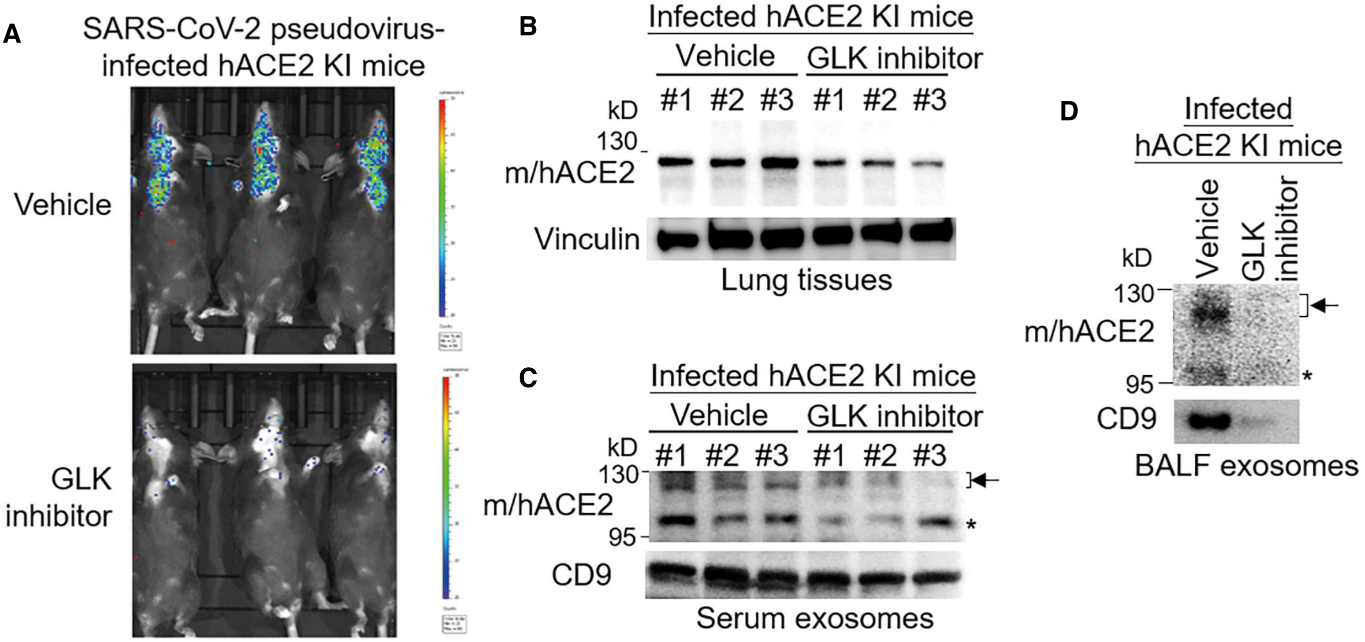

Figure EV5. GLK inhibitor attenuates ACE2 protein levels, ACE2-containing exosomes, and SARS-CoV-2 pseudovirus infection.

A–D Human ACE2 KI mice were intranasally infected with SARS-CoV-2 pseudovirus on Day 0 and Day 1. Mice were also treated with the GLK inhibitor verteporfin on Day 0 after infection. $n = 3$ (biological replicates) per group. Infection efficiencies of SARS-CoV-2 pseudovirus in mouse tissues were measured by IVIS and presented as luminescence counts on Day 2 (A). Immunoblotting of ACE2 and vinculin in the lung tissues of the infected hACE2 KI mice with/without the GLK inhibitor verteporfin (10 μM in 100 μl) treatment (B). Immunoblotting of ACE2 and CD9 in exosomes isolated from the sera (C) and BALF (D) of the infected mice with/without the GLK inhibitor verteporfin (10 μM in 100 μl) treatment. Arrowhead denotes glycosylated ACE2 proteins; asterisk denotes unglycosylated ACE2 proteins.

Data information: WT, wild-type mice; KI, hACE2 knockin mice.

© 2022 The Authors
