# Supplementary material for: SARS‐CoV‐2 spike protein enhances MAP4K3/GLK‐induced ACE2 stability in COVID‐19
Source: EMBO Mol Med. 2022 Jul 27;14(9):e15904. doi: 10.15252/emmm.202215904 (PMC9353388; doi:10.15252/emmm.202215904)
Supplement: Supplementary file 3 — Table EV1 [file EMMM-14-e15904-s007.docx]

CLUSTAL O(1.2.4) multiple sequence alignment

Original-human ACE2 gene sequence

Optimization- codon optimization human ACE2 gene sequence

Original atgtcaagctcttcctggctccttctcagccttgttgctgtaactgctgctcagtccacc 60

Optimization ATGTCATCATCAAGCTGGCTGCTCCTCAGTCTCGTCGCAGTCACCGCCGCACAGTCAACT 60

****** ** ****** ** ***** ** ** ** ** ** ** ** ***** **

Original attgaggaacaggccaagacatttttggacaagtttaaccacgaagccgaagacctgttc 120

Optimization ATCGAAGAACAGGCAAAAACTTTCCTCGACAAGTTCAACCACGAGGCCGAGGATCTGTTT 120

** ** ******** ** ** ** * ******** ******** ***** ** *****

Original tatcaaagttcacttgcttcttggaattataacaccaatattactgaagagaatgtccaa 180

Optimization TACCAGAGCTCCCTGGCTAGCTGGAACTACAACACCAACATCACAGAGGAGAACGTGCAG 180

** ** ** ** ** *** ***** ** ******** ** ** ** ***** ** **

Original aacatgaataatgctggggacaaatggtctgcctttttaaaggaacagtccacacttgcc 240

Optimization AACATGAACAACGCCGGCGACAAGTGGTCCGCTTTCCTGAAGGAGCAGTCTACACTGGCC 240

******** ** ** ** ***** ***** ** ** * ***** ***** ***** ***

Original caaatgtatccactacaagaaattcagaatctcacagtcaagcttcagctgcaggctctt 300

Optimization CAGATGTACCCCCTGCAGGAGATCCAGAACCTGACCGTGAAGCTGCAGCTGCAGGCTCTG 300

** ***** ** ** ** ** ** ***** ** ** ** ***** **************

Original cagcaaaatgggtcttcagtgctctcagaagacaagagcaaacggttgaacacaattcta 360

Optimization CAGCAGAACGGATCTAGCGTGCTGAGCGAGGATAAGTCCAAGCGGCTGAACACAATCCTG 360

***** ** ** *** ***** ** ** *** *** *** ********** **

Original aatacaatgagcaccatctacagtactggaaaagtttgtaacccagataatccacaagaa 420

Optimization AACACCATGTCTACAATCTACAGCACCGGCAAGGTGTGCAACCCCGACAACCCTCAGGAG 420

** ** *** ** ******** ** ** ** ** ** ***** ** ** ** ** **

Original tgcttattacttgaaccaggtttgaatgaaataatggcaaacagtttagactacaatgag 480

Optimization TGTCTGCTGCTGGAGCCTGGACTGAACGAGATCATGGCCAACTCCCTGGATTACAACGAG 480

** * * ** ** ** ** **** ** ** ***** *** * ** ***** ***

Original aggctctgggcttgggaaagctggagatctgaggtcggcaagcagctgaggccattatat 540

Optimization AGGCTGTGGGCTTGGGAGAGCTGGAGATCCGAAGTGGGCAAGCAGCTGCGGCCACTGTAC 540

***** *********** *********** ** ** ************ ***** * **

Original gaagagtatgtggtcttgaaaaatgagatggcaagagcaaatcattatgaggactatggg 600

Optimization GAGGAGTACGTGGTGCTGAAGAACGAGATGGCCCGCGCTAACCACTACGAGGACTACGGC 600

** ***** ***** **** ** ******** * ** ** ** ** ******** **

Original gattattggagaggagactatgaagtaaatggggtagatggctatgactacagccgcggc 660

Optimization GATTACTGGAGGGGAGACTACGAGGTGAACGGCGTGGACGGATACGATTACAGCAGAGGC 660

***** ***** ******** ** ** ** ** ** ** ** ** ** ****** * ***

Original cagttgattgaagatgtggaacatacctttgaagagattaaaccattatatgaacatctt 720

Optimization CAGCTGATCGAGGATGTGGAGCACACATTCGAGGAGATCAAGCCTCTGTACGAGCACCTG 720

*** **** ** ******** ** ** ** ** ***** ** ** * ** ** ** **

Original catgcctatgtgagggcaaagttgatgaatgcctatccttcctatatcagtccaattgga 780

Optimization CACGCCTACGTGCGGGCTAAGCTGATGAACGCCTACCCATCTTACATCAGCCCAATCGGA 780

** ***** *** **** *** ******* ***** ** ** ** ***** ***** ***

Original tgcctccctgctcatttgcttggtgatatgtggggtagattttggacaaatctgtactct 840

Optimization TGCCTGCCTGCTCACCTGCTGGGCGACATGTGGGGACGCTTCTGGACAAACCTGTACTCC 840

***** ******** **** ** ** ******** * ** ******** ********

Original ttgacagttccctttggacagaaaccaaacatagatgttactgatgcaatggtggaccag 900

Optimization CTGACCGTGCCATTTGGACAGAAGCCCAACATCGACGTGACCGATGCCATGGTGGACCAG 900

**** ** ** *********** ** ***** ** ** ** ***** ************

Original gcctgggatgcacagagaatattcaaggaggccgagaagttctttgtatctgttggtctt 960

Optimization GCCTGGGATGCTCAGAGGATCTTCAAGGAGGCTGAGAAGTTCTTCGTGAGCGTGGGCCTG 960

*********** ***** ** *********** *********** ** ** ** **

Original cctaatatgactcaaggattctgggaaaattccatgctaacggacccaggaaatgttcag 1020

Optimization CCTAACATGACACAGGGATTTTGGGAGAACTCTATGCTGACCGACCCAGGCAACGTGCAG 1020

***** ***** ** ***** ***** ** ** ***** ** ******** ** ** ***

Original aaagcagtctgccatcccacagcttgggacctggggaagggcgacttcaggatccttatg 1080

Optimization AAGGCCGTGTGCCACCCCACAGCTTGGGACCTGGGCAAGGGAGATTTCAGAATCCTGATG 1080

** ** ** ***** ******************** ***** ** ***** ***** ***

Original tgcacaaaggtgacaatggacgacttcctgacagctcatcatgagatggggcatatccag 1140

Optimization TGCACCAAGGTGACAATGGACGATTTTCTGACCGCCCACCACGAGATGGGACACATCCAG 1140

***** ***************** ** ***** ** ** ** ******** ** ******

Original tatgatatggcatatgctgcacaaccttttctgctaagaaatggagctaatgaaggattc 1200

Optimization TACGATATGGCTTACGCCGCTCAGCCATTCCTGCTGCGGAACGGCGCCAACGAGGGATTT 1200

** ******** ** ** ** ** ** ** ***** * ** ** ** ** ** *****

Original catgaagctgttggggaaatcatgtcactttctgcagccacacctaagcatttaaaatcc 1260

Optimization CACGAGGCTGTGGGCGAGATCATGAGCCTGTCCGCCGCTACACCTAAGCACCTGAAGTCT 1260

** ** ***** ** ** ****** ** ** ** ** *********** * ** **

Original attggtcttctgtcacccgattttcaagaagacaatgaaacagaaataaacttcctgctc 1320

Optimization ATCGGACTGCTGAGCCCAGACTTCCAGGAGGATAACGAGACAGAGATCAACTTTCTGCTG 1320

** ** ** *** ** ** ** ** ** ** ** ** ***** ** ***** *****

Original aaacaagcactcacgattgttgggactctgccatttacttacatgttagagaagtggagg 1380

Optimization AAGCAGGCCCTGACCATCGTGGGCACACTGCCCTTCACCTACATGCTGGAGAAGTGGAGA 1380

** ** ** ** ** ** ** ** ** ***** ** ** ****** * ***********

Original tggatggtctttaaaggggaaattcccaaagaccagtggatgaaaaagtggtgggagatg 1440

Optimization TGGATGGTGTTTAAGGGAGAGATCCCTAAGGACCAGTGGATGAAGAAGTGGTGGGAGATG 1440

******** ***** ** ** ** ** ** ************** ***************

Original aagcgagagatagttggggtggtggaacctgtgccccatgatgaaacatactgtgacccc 1500

Optimization AAGCGGGAGATCGTGGGCGTGGTGGAGCCTGTGCCACACGACGAGACATACTGTGATCCA 1500

***** ***** ** ** ******** ******** ** ** ** *********** **

Original gcatctctgttccatgtttctaatgattactcattcattcgatattacacaaggaccctt 1560

Optimization GCCTCCCTGTTCCACGTGTCCAACGACTACTCTTTTATCAGGTACTACACCAGAACACTG 1560

** ** ******** ** ** ** ** ***** ** ** * ** ***** ** ** **

Original taccaattccagtttcaagaagcactttgtcaagcagctaaacatgaaggccctctgcac 1620

Optimization TACCAGTTCCAGTTTCAGGAGGCCCTGTGCCAGGCTGCTAAGCACGAGGGACCACTGCAC 1620

***** *********** ** ** ** ** ** ** ***** ** ** ** ** ******

Original aaatgtgacatctcaaactctacagaagctggacagaaactgttcaatatgctgaggctt 1680

Optimization AAGTGTGATATCTCTAACAGCACAGAGGCCGGCCAGAAGCTGTTCAACATGCTGCGCCTG 1680

** ***** ***** *** ***** ** ** ***** ******** ****** * **

Original ggaaaatcagaaccctggaccctagcattggaaaatgttgtaggagcaaagaacatgaat 1740

Optimization GGAAAGTCTGAGCCCTGGACCCTGGCCCTGGAGAACGTGGTGGGAGCTAAGAACATGAAC 1740

***** ** ** *********** ** **** ** ** ** ***** ***********

Original gtaaggccactgctcaactactttgagcccttatttacctggctgaaagaccagaacaag 1800

Optimization GTGAGGCCTCTGCTGAACTACTTCGAGCCACTGTTTACATGGCTGAAGGACCAGAACAAG 1800

** ***** ***** ******** ***** * ***** ******** ************

Original aattcttttgtgggatggagtaccgactggagtccatatgcagaccaaagcatcaaagtg 1860

Optimization AACAGCTTCGTGGGCTGGAGCACCGACTGGTCCCCATACGCCGATCAGTCCATCAAGGTG 1860

** ** ***** ***** ********* ***** ** ** ** ****** ***

Original aggataagcctaaaatcagctcttggagataaagcatatgaatggaacgacaatgaaatg 1920

Optimization AGGATCTCCCTGAAGTCTGCCCTGGGAGACAAGGCTTACGAGTGGAACGATAACGAGATG 1920

***** *** ** ** ** ** ***** ** ** ** ** ******** ** ** ***

Original tacctgttccgatcatctgttgcatatgctatgaggcagtactttttaaaagtaaaaaat 1980

Optimization TACCTGTTCCGCTCCTCTGTGGCCTACGCTATGAGGCAGTACTTCCTGAAGGTGAAGAAC 1980

*********** ** ***** ** ** ***************** * ** ** ** **

Original cagatgattctttttggggaggaggatgtgcgagtggctaatttgaaaccaagaatctcc 2040

Optimization CAGATGATCCTGTTTGGAGAGGAGGACGTGCGGGTGGCTAACCTGAAGCCACGCATCTCT 2040

******** ** ***** ******** ***** ******** **** *** * *****

Original tttaatttctttgtcactgcacctaaaaatgtgtctgatatcattcctagaactgaagtt 2100

Optimization TTTAACTTCTTTGTGACAGCTCCCAAGAACGTGAGCGATATCATCCCTAGAACCGAGGTG 2100

***** ******** ** ** ** ** ** *** ******** ******** ** **

Original gaaaaggccatcaggatgtcccggagccgtatcaatgatgctttccgtctgaatgacaac 2160

Optimization GAGAAGGCCATCCGGATGTCCAGGTCTAGAATCAACGACGCTTTCCGCCTGAACGATAAC 2160

** ********* ******** ** * ***** ** ******** ***** ** ***

Original agcctagagtttctggggatacagccaacacttggacctcctaaccagccccctgtttcc 2220

Optimization TCTCTGGAGTTTCTGGGCATCCAGCCCACACTGGGACCCCCTAACCAGCCACCCGTGAGC 2220

** *********** ** ***** ***** ***** *********** ** ** *

Original atatggctgattgtttttggagttgtgatgggagtgatagtggttggcattgtcatcctg 2280

Optimization ATCTGGCTGATCGTGTTCGGCGTGGTCATGGGAGTGATCGTGGTGGGCATCGTGATCCTG 2280

** ******** ** ** ** ** ** *********** ***** ***** ** ******

Original atcttcactgggatcagagatcggaagaagaaaaataaagcaagaagtggagaaaatcct 2340

Optimization ATCTTTACCGGCATCAGGGACAGGAAGAAGAAGAACAAGGCCAGAAGCGGCGAGAACCCT 2340

***** ** ** ***** ** ********** ** ** ** ***** ** ** ** ***

Original tatgcctccatcgatattagcaaaggagaaaataatccaggattccaaaacactgatgat 2400

Optimization TACGCTTCCATCGATATCTCTAAAGGAGAGAACAATCCAGGTTTCCAGAACACCGACGAT 2400

** ** *********** ******** ** ******** ***** ***** ** ***

Original gttcagacctccttt--- 2415

Optimization GTCCAGACCAGTTTTTGA 2418

** ****** ***

Amino acid sequence

MSSSSWLLLSLVAVTAAQSTIEEQAKTFLDKFNHEAEDLFYQSSLASWNYNTNITEENVQNMNNAGDKWSAFLKEQSTLAQMYPLQEIQNLTVKLQLQALQQNGSSVLSEDKSKRLNTILNTMSTIYSTGKVCNPDNPQECLLLEPGLNEIMANSLDYNERLWAWESWRSEVGKQLRPLYEEYVVLKNEMARANHYEDYGDYWRGDYEVNGVDGYDYSRGQLIEDVEHTFEEIKPLYEHLHAYVRAKLMNAYPSYISPIGCLPAHLLGDMWGRFWTNLYSLTVPFGQKPNIDVTDAMVDQAWDAQRIFKEAEKFFVSVGLPNMTQGFWENSMLTDPGNVQKAVCHPTAWDLGKGDFRILMCTKVTMDDFLTAHHEMGHIQYDMAYAAQPFLLRNGANEGFHEAVGEIMSLSAATPKHLKSIGLLSPDFQEDNETEINFLLKQALTIVGTLPFTYMLEKWRWMVFKGEIPKDQWMKKWWEMKREIVGVVEPVPHDETYCDPASLFHVSNDYSFIRYYTRTLYQFQFQEALCQAAKHEGPLHKCDISNSTEAGQKLFNMLRLGKSEPWTLALENVVGAKNMNVRPLLNYFEPLFTWLKDQNKNSFVGWSTDWSPYADQSIKVRISLKSALGDKAYEWNDNEMYLFRSSVAYAMRQYFLKVKNQMILFGEEDVRVANLKPRISFNFFVTAPKNVSDIIPRTEVEKAIRMSRSRINDAFRLNDNSLEFLGIQPTLGPPNQPPVSIWLIVFGVVMGVIVVGIVILIFTGIRDRKKKNKARSGENPYASIDISKGENNPGFQNTDDVQTSF*

Original human ACE2 gene sequence

atgtcaagctcttcctggctccttctcagccttgttgctgtaactgctgctcagtccaccattgaggaacaggccaagacatttttggacaagtttaaccacgaagccgaagacctgttctatcaaagttcacttgcttcttggaattataacaccaatattactgaagagaatgtccaaaacatgaataatgctggggacaaatggtctgcctttttaaaggaacagtccacacttgcccaaatgtatccactacaagaaattcagaatctcacagtcaagcttcagctgcaggctcttcagcaaaatgggtcttcagtgctctcagaagacaagagcaaacggttgaacacaattctaaatacaatgagcaccatctacagtactggaaaagtttgtaacccagataatccacaagaatgcttattacttgaaccaggtttgaatgaaataatggcaaacagtttagactacaatgagaggctctgggcttgggaaagctggagatctgaggtcggcaagcagctgaggccattatatgaagagtatgtggtcttgaaaaatgagatggcaagagcaaatcattatgaggactatggggattattggagaggagactatgaagtaaatggggtagatggctatgactacagccgcggccagttgattgaagatgtggaacatacctttgaagagattaaaccattatatgaacatcttcatgcctatgtgagggcaaagttgatgaatgcctatccttcctatatcagtccaattggatgcctccctgctcatttgcttggtgatatgtggggtagattttggacaaatctgtactctttgacagttccctttggacagaaaccaaacatagatgttactgatgcaatggtggaccaggcctgggatgcacagagaatattcaaggaggccgagaagttctttgtatctgttggtcttcctaatatgactcaaggattctgggaaaattccatgctaacggacccaggaaatgttcagaaagcagtctgccatcccacagcttgggacctggggaagggcgacttcaggatccttatgtgcacaaaggtgacaatggacgacttcctgacagctcatcatgagatggggcatatccagtatgatatggcatatgctgcacaaccttttctgctaagaaatggagctaatgaaggattccatgaagctgttggggaaatcatgtcactttctgcagccacacctaagcatttaaaatccattggtcttctgtcacccgattttcaagaagacaatgaaacagaaataaacttcctgctcaaacaagcactcacgattgttgggactctgccatttacttacatgttagagaagtggaggtggatggtctttaaaggggaaattcccaaagaccagtggatgaaaaagtggtgggagatgaagcgagagatagttggggtggtggaacctgtgccccatgatgaaacatactgtgaccccgcatctctgttccatgtttctaatgattactcattcattcgatattacacaaggaccctttaccaattccagtttcaagaagcactttgtcaagcagctaaacatgaaggccctctgcacaaatgtgacatctcaaactctacagaagctggacagaaactgttcaatatgctgaggcttggaaaatcagaaccctggaccctagcattggaaaatgttgtaggagcaaagaacatgaatgtaaggccactgctcaactactttgagcccttatttacctggctgaaagaccagaacaagaattcttttgtgggatggagtaccgactggagtccatatgcagaccaaagcatcaaagtgaggataagcctaaaatcagctcttggagataaagcatatgaatggaacgacaatgaaatgtacctgttccgatcatctgttgcatatgctatgaggcagtactttttaaaagtaaaaaatcagatgattctttttggggaggaggatgtgcgagtggctaatttgaaaccaagaatctcctttaatttctttgtcactgcacctaaaaatgtgtctgatatcattcctagaactgaagttgaaaaggccatcaggatgtcccggagccgtatcaatgatgctttccgtctgaatgacaacagcctagagtttctggggatacagccaacacttggacctcctaaccagccccctgtttccatatggctgattgtttttggagttgtgatgggagtgatagtggttggcattgtcatcctgatcttcactgggatcagagatcggaagaagaaaaataaagcaagaagtggagaaaatccttatgcctccatcgatattagcaaaggagaaaataatccaggattccaaaacactgatgatgttcagacctccttt

Codon optimization sequence

ATGTCATCATCAAGCTGGCTGCTCCTCAGTCTCGTCGCAGTCACCGCCGCACAGTCAACTATCGAAGAACAGGCAAAAACTTTCCTCGACAAGTTCAACCACGAGGCCGAGGATCTGTTTTACCAGAGCTCCCTGGCTAGCTGGAACTACAACACCAACATCACAGAGGAGAACGTGCAGAACATGAACAACGCCGGCGACAAGTGGTCCGCTTTCCTGAAGGAGCAGTCTACACTGGCCCAGATGTACCCCCTGCAGGAGATCCAGAACCTGACCGTGAAGCTGCAGCTGCAGGCTCTGCAGCAGAACGGATCTAGCGTGCTGAGCGAGGATAAGTCCAAGCGGCTGAACACAATCCTGAACACCATGTCTACAATCTACAGCACCGGCAAGGTGTGCAACCCCGACAACCCTCAGGAGTGTCTGCTGCTGGAGCCTGGACTGAACGAGATCATGGCCAACTCCCTGGATTACAACGAGAGGCTGTGGGCTTGGGAGAGCTGGAGATCCGAAGTGGGCAAGCAGCTGCGGCCACTGTACGAGGAGTACGTGGTGCTGAAGAACGAGATGGCCCGCGCTAACCACTACGAGGACTACGGCGATTACTGGAGGGGAGACTACGAGGTGAACGGCGTGGACGGATACGATTACAGCAGAGGCCAGCTGATCGAGGATGTGGAGCACACATTCGAGGAGATCAAGCCTCTGTACGAGCACCTGCACGCCTACGTGCGGGCTAAGCTGATGAACGCCTACCCATCTTACATCAGCCCAATCGGATGCCTGCCTGCTCACCTGCTGGGCGACATGTGGGGACGCTTCTGGACAAACCTGTACTCCCTGACCGTGCCATTTGGACAGAAGCCCAACATCGACGTGACCGATGCCATGGTGGACCAGGCCTGGGATGCTCAGAGGATCTTCAAGGAGGCTGAGAAGTTCTTCGTGAGCGTGGGCCTGCCTAACATGACACAGGGATTTTGGGAGAACTCTATGCTGACCGACCCAGGCAACGTGCAGAAGGCCGTGTGCCACCCCACAGCTTGGGACCTGGGCAAGGGAGATTTCAGAATCCTGATGTGCACCAAGGTGACAATGGACGATTTTCTGACCGCCCACCACGAGATGGGACACATCCAGTACGATATGGCTTACGCCGCTCAGCCATTCCTGCTGCGGAACGGCGCCAACGAGGGATTTCACGAGGCTGTGGGCGAGATCATGAGCCTGTCCGCCGCTACACCTAAGCACCTGAAGTCTATCGGACTGCTGAGCCCAGACTTCCAGGAGGATAACGAGACAGAGATCAACTTTCTGCTGAAGCAGGCCCTGACCATCGTGGGCACACTGCCCTTCACCTACATGCTGGAGAAGTGGAGATGGATGGTGTTTAAGGGAGAGATCCCTAAGGACCAGTGGATGAAGAAGTGGTGGGAGATGAAGCGGGAGATCGTGGGCGTGGTGGAGCCTGTGCCACACGACGAGACATACTGTGATCCAGCCTCCCTGTTCCACGTGTCCAACGACTACTCTTTTATCAGGTACTACACCAGAACACTGTACCAGTTCCAGTTTCAGGAGGCCCTGTGCCAGGCTGCTAAGCACGAGGGACCACTGCACAAGTGTGATATCTCTAACAGCACAGAGGCCGGCCAGAAGCTGTTCAACATGCTGCGCCTGGGAAAGTCTGAGCCCTGGACCCTGGCCCTGGAGAACGTGGTGGGAGCTAAGAACATGAACGTGAGGCCTCTGCTGAACTACTTCGAGCCACTGTTTACATGGCTGAAGGACCAGAACAAGAACAGCTTCGTGGGCTGGAGCACCGACTGGTCCCCATACGCCGATCAGTCCATCAAGGTGAGGATCTCCCTGAAGTCTGCCCTGGGAGACAAGGCTTACGAGTGGAACGATAACGAGATGTACCTGTTCCGCTCCTCTGTGGCCTACGCTATGAGGCAGTACTTCCTGAAGGTGAAGAACCAGATGATCCTGTTTGGAGAGGAGGACGTGCGGGTGGCTAACCTGAAGCCACGCATCTCTTTTAACTTCTTTGTGACAGCTCCCAAGAACGTGAGCGATATCATCCCTAGAACCGAGGTGGAGAAGGCCATCCGGATGTCCAGGTCTAGAATCAACGACGCTTTCCGCCTGAACGATAACTCTCTGGAGTTTCTGGGCATCCAGCCCACACTGGGACCCCCTAACCAGCCACCCGTGAGCATCTGGCTGATCGTGTTCGGCGTGGTCATGGGAGTGATCGTGGTGGGCATCGTGATCCTGATCTTTACCGGCATCAGGGACAGGAAGAAGAAGAACAAGGCCAGAAGCGGCGAGAACCCTTACGCTTCCATCGATATCTCTAAAGGAGAGAACAATCCAGGTTTCCAGAACACCGACGATGTCCAGACCAGTTTTTGA

Genotyping primer sequence

mhACE2-569-F

GCCACTGTACGAGGAGTAC

mhACE2-659-R

CCTCTGCTGTAATCGTATCCG
