# Supplementary figures and images for: SARS‐CoV‐2 spike protein enhances MAP4K3/GLK‐induced ACE2 stability in COVID‐19
Source: EMBO Mol Med. 2022 Jul 27;14(9):e15904. doi: 10.15252/emmm.202215904 (PMC9353388; doi:10.15252/emmm.202215904)

Figure 2A

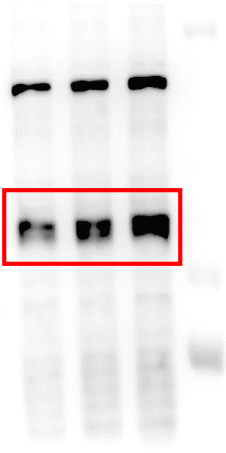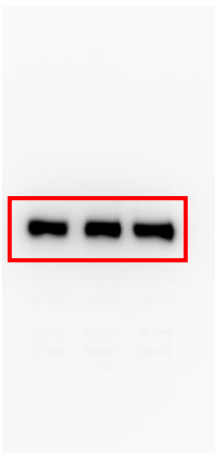

Figure 2C

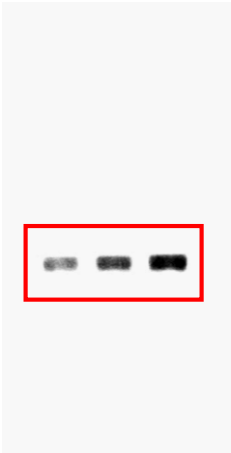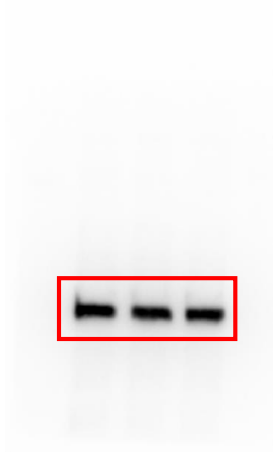

Supplement: Supplementary file 5 — Source Data for Figure 2 [file EMMM-14-e15904-s003.pdf]

Figure 3B

---

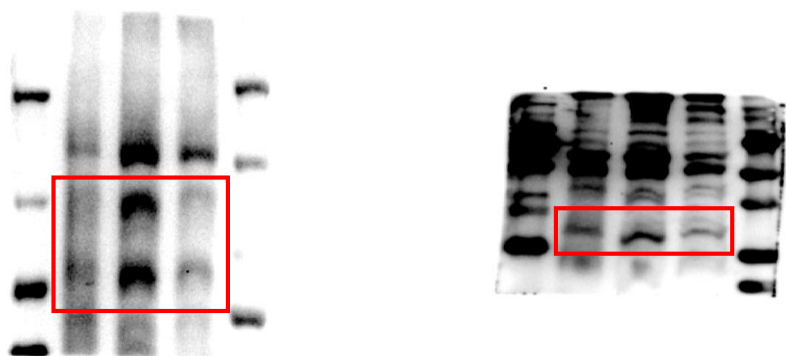

Figure 3C

---

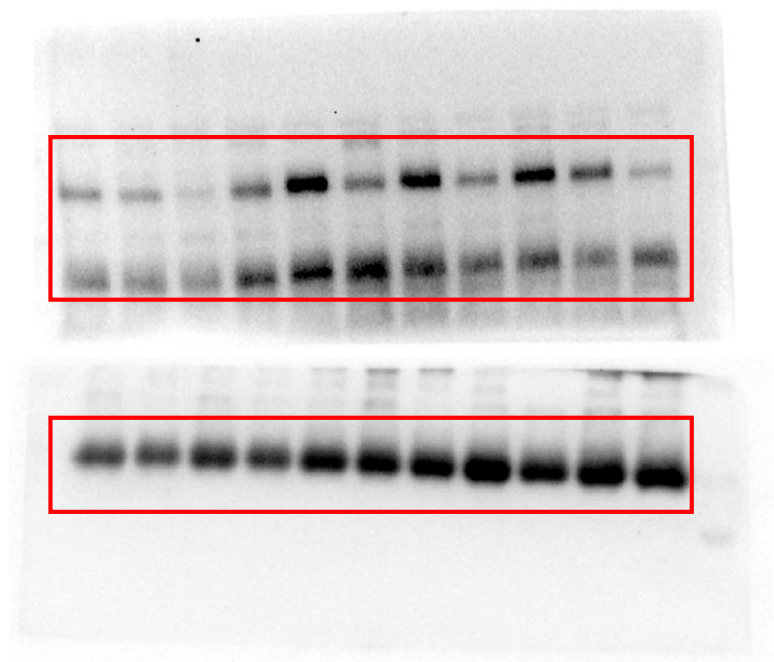

Supplement: Supplementary file 6 — Source Data for Figure 3 [file EMMM-14-e15904-s004.pdf]

Figure 4G

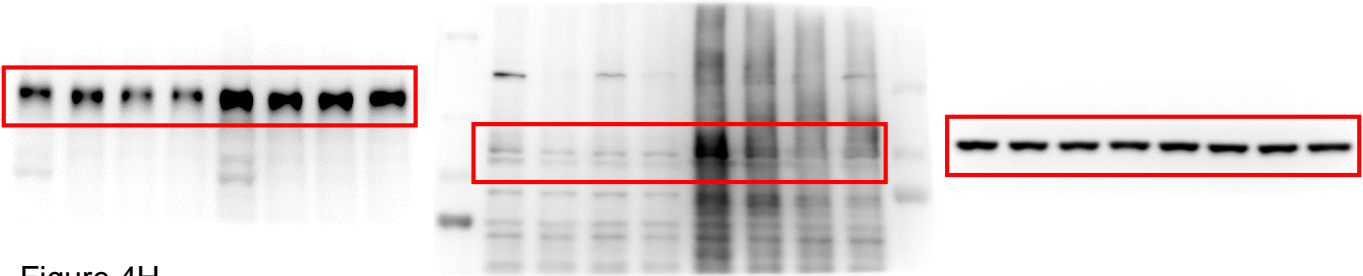

Figure 4H

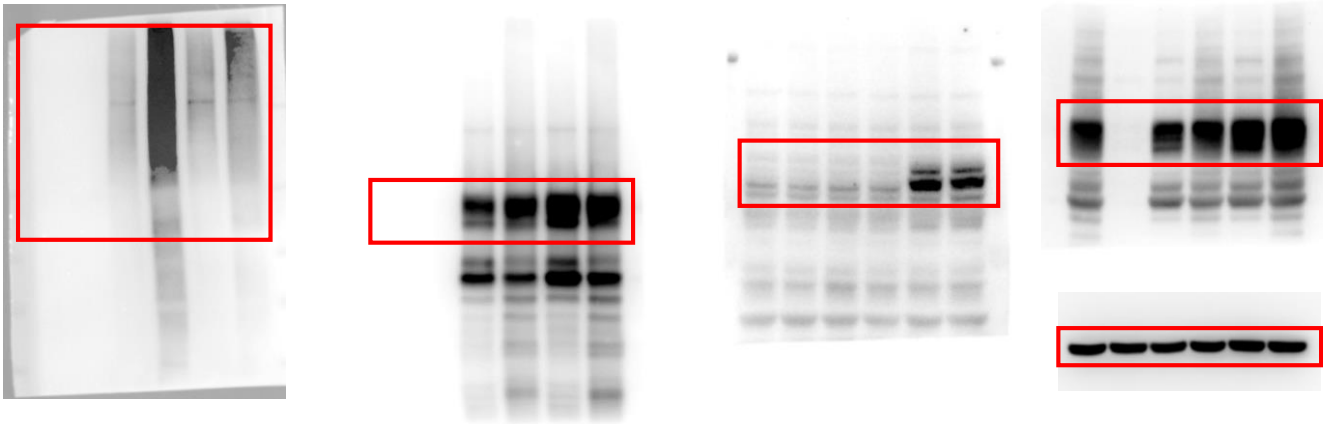

Supplement: Supplementary file 7 — Source Data for Figure 4 [file EMMM-14-e15904-s006.pdf]

Figure 5A

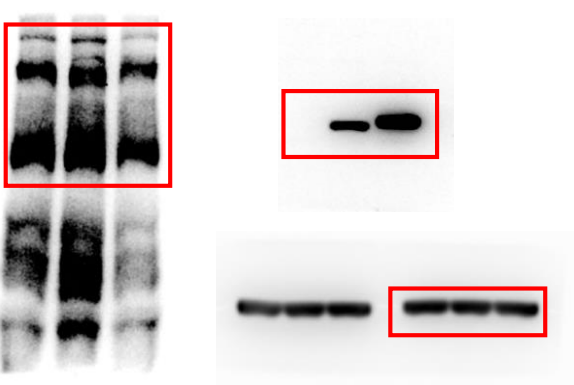

Figure 5B

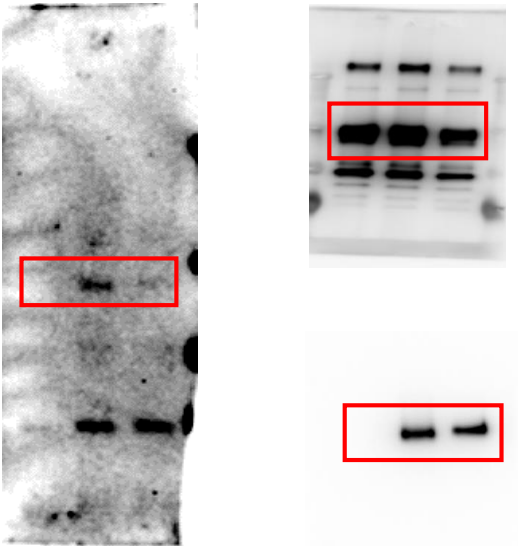

Figure 5E

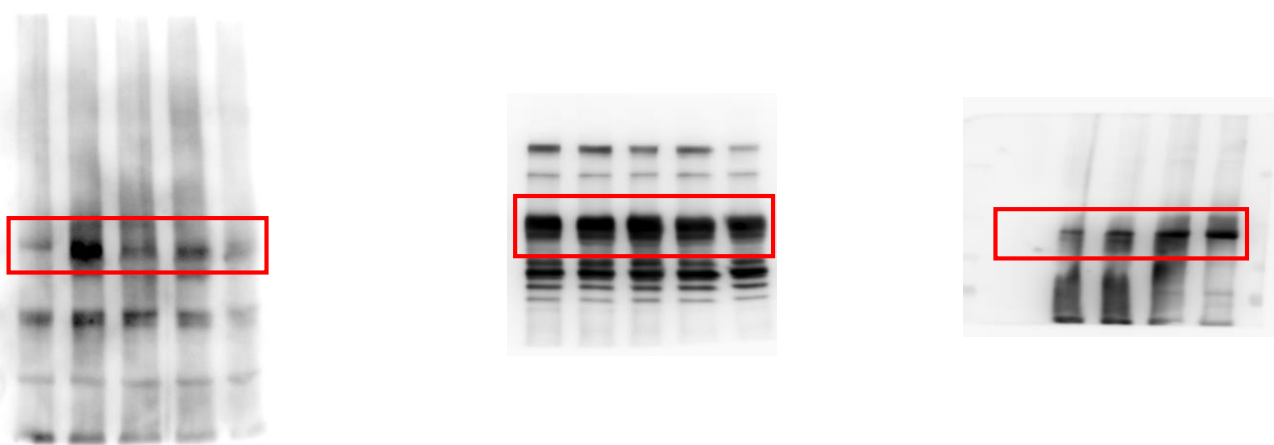

Figure 5F

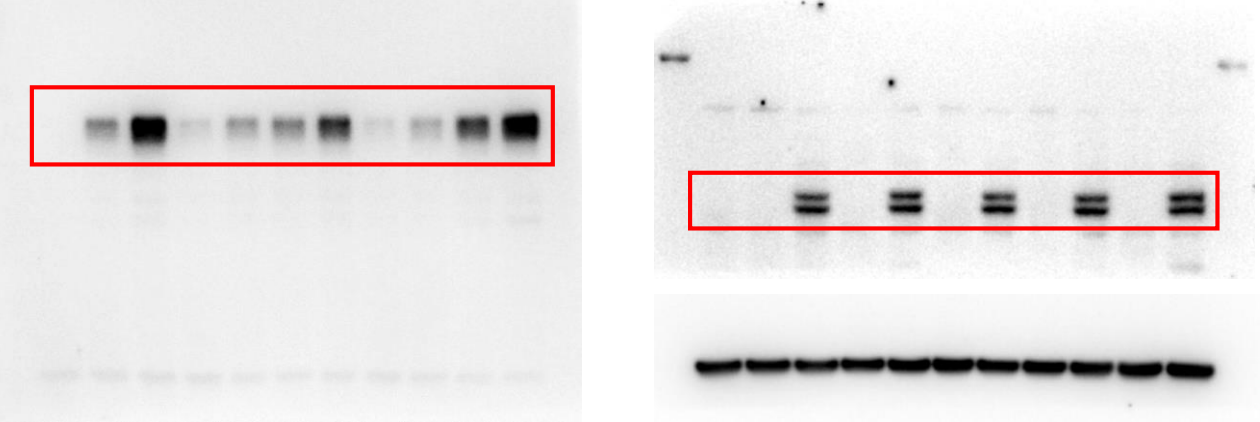

Figure 5G

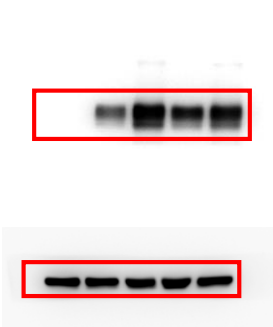

Figure 5H

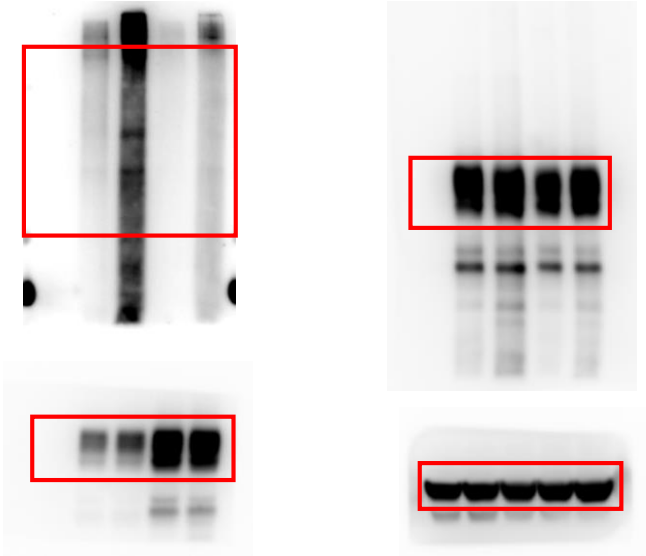

Supplement: Supplementary file 8 — Source Data for Figure 5 [file EMMM-14-e15904-s009.pdf]

Figure 6B

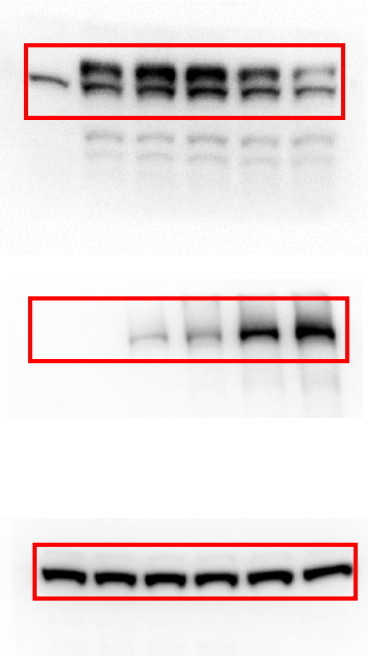

Figure 6C

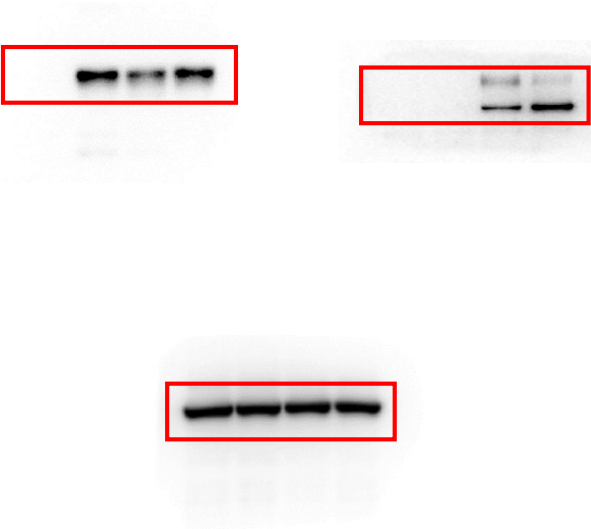

Figure 6D

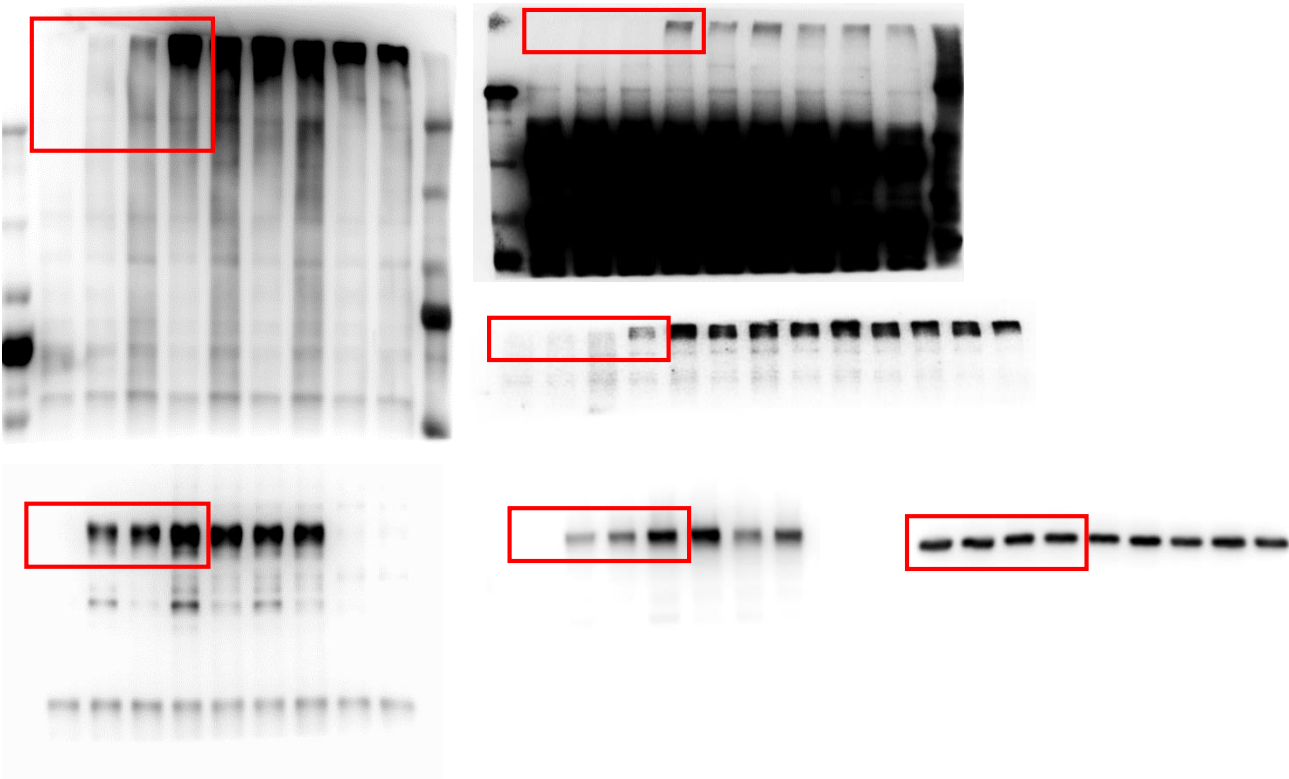

Figure 6G

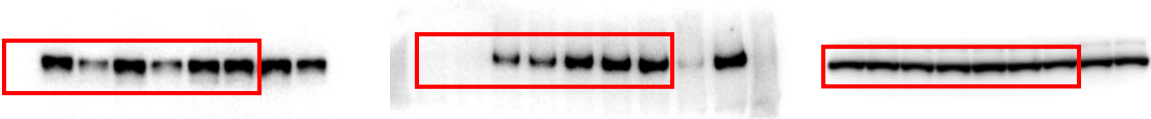

Figure 6H

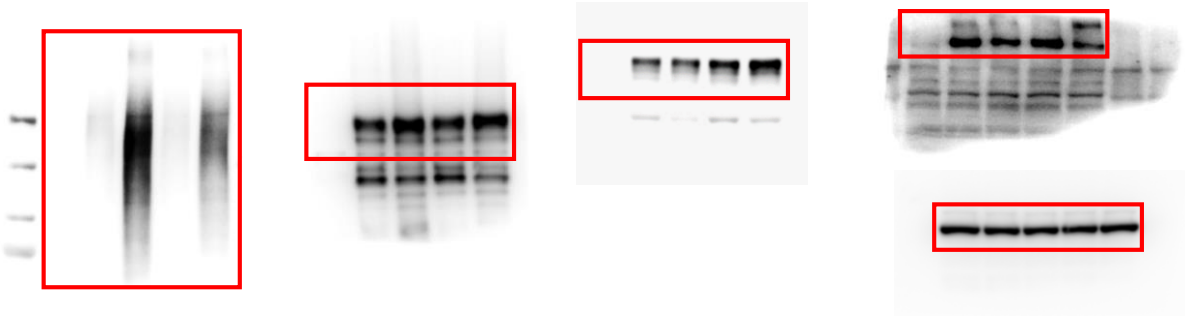

Supplement: Supplementary file 9 — Source Data for Figure 6 [file EMMM-14-e15904-s011.pdf]

Figure 7C

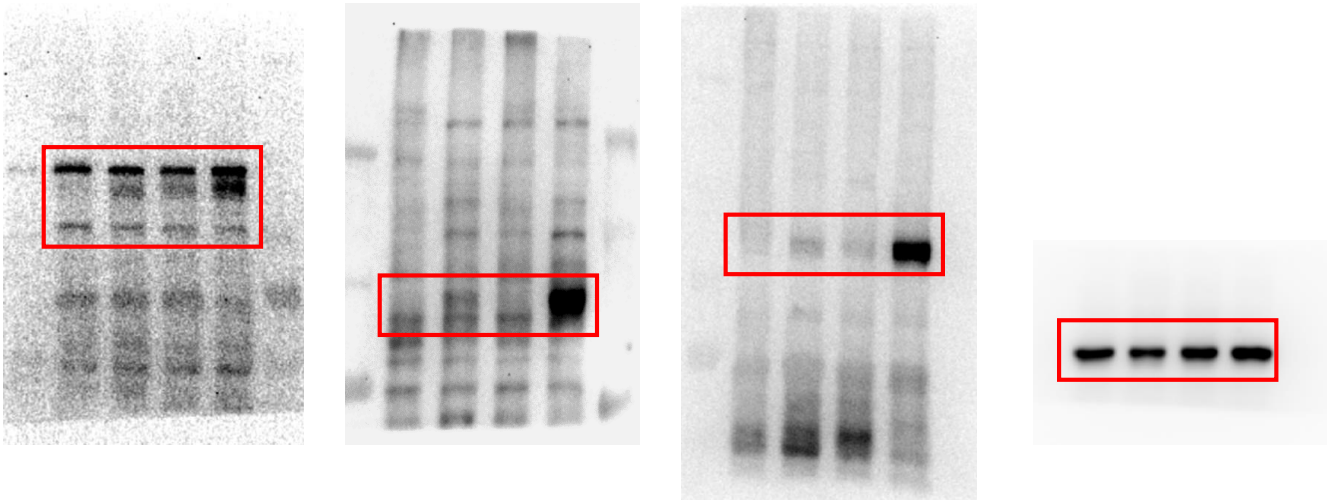

Figure 7E

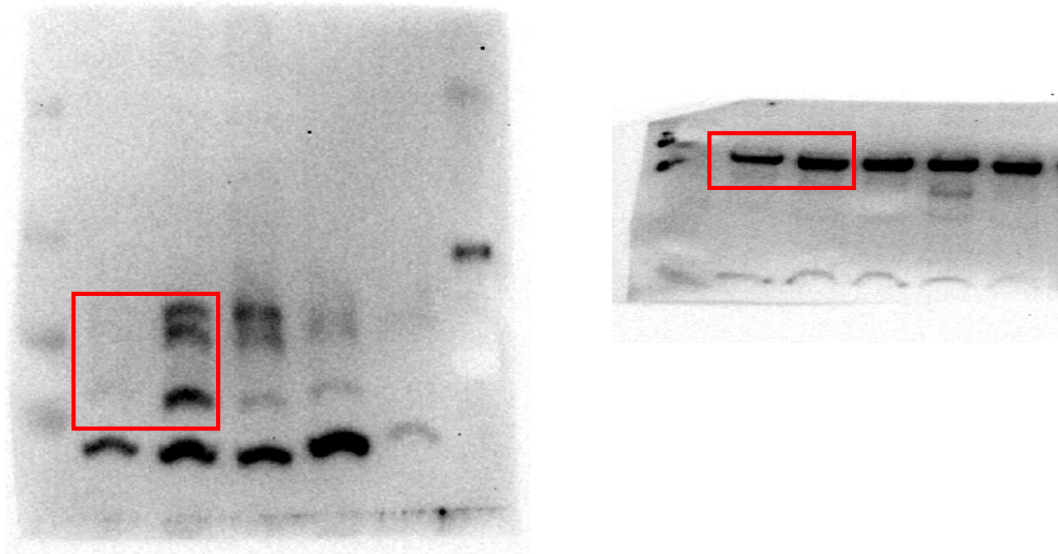

Supplement: Supplementary file 10 — Source Data for Figure 7 [file EMMM-14-e15904-s001.pdf]
