# Supplementary material for: SARS‐CoV‐2 spike protein enhances MAP4K3/GLK‐induced ACE2 stability in COVID‐19
Source: EMBO Mol Med. 2022 Jul 27;14(9):e15904. doi: 10.15252/emmm.202215904 (PMC9353388; doi:10.15252/emmm.202215904)
Supplement: Supplementary file 6 [file EMMM-14-e15904-s008.pdf]

EMBO Molecular Medicine

SARS-CoV-2 spike protein enhances MAP4K3/GLK-induced ACE2 stability in COVID-19

Huai-Chia Chuang, Chia-Hsin Hsueh, Pu-Ming Hsu, Rou-Huei Huang, Ching-Yi Tsai, Nai-Hsiang Chung, Yen-Hung Chow, and Tse-Hua Tan
DOI: 10.15252/emmm.202215904

Corresponding author(s): Tse-Hua Tan (ttan@nhri.edu.tw) , Huai-Chia Chuang (cinth@nhri.edu.tw), Yen-Hung Chow (choeyenh@nhri.edu.tw)

Review Timeline:

Submission Date:	17th Feb 22
Editorial Decision:	24th Mar 22
Revision Received:	22nd Jun 22
Editorial Decision:	4th Jul 22
Revision Received:	11th Jul 22
Accepted:	12th Jul 22

Editor: Zeljko Durdevic

Transaction Report:

(Note: With the exception of the correction of typographical or spelling errors that could be a source of ambiguity, letters and reports are not edited. Depending on transfer agreements, referee reports obtained elsewhere may or may not be included in this compilation. Referee reports are anonymous unless the Referee chooses to sign their reports.)

24th Mar 2022

Dear Prof. Tan,

Thank you for the submission of your manuscript to EMBO Molecular Medicine. We have now received feedback from the three reviewers who agreed to evaluate your manuscript. As you will see from the reports below, the referees acknowledge the interest of the study but also raise important concerns that should be addressed in a major revision.

Further consideration of a revision that addresses reviewers' concerns in full will entail a second round of review. EMBO Molecular Medicine encourages a single round of revision only and therefore, acceptance or rejection of the manuscript will depend on the completeness of your responses included in the next, final version of the manuscript. For this reason, and to save you from any frustrations in the end, I would strongly advise against returning an incomplete revision.

We would welcome the submission of a revised version within three months for further consideration. Please let us know if you require longer to complete the revision.

Please use this link to login to the manuscript system and submit your revision: https://embomolmed.msubmit.net/cgi-bin/main.plex

I look forward to receiving your revised manuscript.

Yours sincerely,

Zeljko Durdevic

Zeljko Durdevic
Editor
EMBO Molecular Medicine

.

***** Reviewer's comments *****

Referee #1 (Comments on Novelty/Model System for Author):

scRNA-seq and proteomics analyses using four COVID-19 patient cohorts including one from Taiwan.

The authors found that both protein levels and mRNA levels of GLK were increased in pseudovirus-infected HCC827 lung epithelial cells, as well as GLK levels increasing with the spike protein.

They also utilized UBR4 overexpression which enhanced Lys48-linked ubiquitination of ACE2 leading to proteosmal degradation of ACE2.

Referee #1 (Remarks for Author):

The current manuscript titled "SARS-CoV-2 spike protein enhances MAP4K3-induced ACE2 stability in COVID-19" describes whether GLK is involved in COVID-19 pathogenesis by scRNA-seq and proteomics analyses using four COVID-19 patient cohorts from Taiwan. Interestingly, the results suggest that GLK stabilizes ACE2 proteins in epithelial cells, enhancing SARS-CoV-2 infection susceptibility of epithelial cells. More specifically, the authors found that both protein levels and mRNA levels of GLK were increased in pseudovirus-infected HCC827 lung epithelial cells, as well as GLK levels increasing with the spike protein. They also found increased levels of the exosome marker CD63 in serum exosomes suggesting an enhancement of exosome release in COVID-19 patients. In situ proximity ligation assays (PLA) with ACE2 showed PLA signals in cells co-transfected with ACE2 and Flag-GLK, suggested a direct interaction between ACE2 and GLK. They also utilized UBR4 overexpression which enhanced Lys48-linked ubiquitination of ACE2 leading to proteasomal degradation of ACE2. Specificity controls included use of ACE2 (K26R), ACE2 (K112R), and ACE2 (K114R) and ACE2 (K94R) mutation resistant to UBR4-induced ACE2 degradation. Overall, this is an interesting and novel manuscript, however there are few minor concerns that include:

1. Have the authors performed any analysis on the exosomal contents? Are there any proteins related to the mitochondrial pathway? Any functional analysis of the exosomes on recipient cells?
2. Data in Figure 7 is very interesting, but somewhat overwhelming! Can the authors break up this figure into two and explain the results better in the result and discussion sections with more depth?

Referee #2 (Remarks for Author):

In this manuscript, Chuang et al found that SARS-CoV-2 infection, particularly the spike protein, can induce the GLK expression. In addition, GLK can induce release of exosome with ACE2 and stabilize ACE2 by reducing ubiquitination, thus promoting the SARS-CoV-2 infection. It is an interesting and novel pathway of SARS-CoV-2 pathogenesis, which will attract wide attentions from virologist. This pathway can also serve as a therapeutic target for COVID-19 treatment.
This manuscript is well organized. The references are correctly cited. The methods are well described. However, some modifications should be made to further improve the solidarity of the conclusion.

1. In figure 1C and D, and EV2, the figure legend indicates percentage while the figures show cell counts. The increase in GLK+ cell count cannot support that GLK expression was induced by SARS-CoV-2 infection since the total cell count in BLAF in SARS-CoV-2 patients are increased. So the percentage should be used here. Besides, the statistical analysis should use ANOVA instead of t-test since there are more than 2 groups. Same problem is also in figure 4I.
2. To test whether SARS-CoV-2 infection results in GLK overexpression in lung epithelial cells, the author used SARS-CoV-2

pseudovirus. A control pseudovirus with other viral surface protein should be added to see whether this GLK overexpression is specifically induced by SARS-CoV-2.
3. Similarly, in figure 3F, another psudovirus should be used here to confirm whether the enhenced viral infection results from increased ACE2 level or other non-specific mechanism, such as enhanced endocytosis. Same problem is also in figure 4I.
4. In figure 4D, why the lower bands of ACE2 contains 3 bands.
5. The quality of Figure 5A is poor.

Minors:
1. In page 5, the author mentioned figure 1E and F, but no figure 1E and F in the figure section.
2. In methods section -- SARS-CoV-2 pseudovirus infection mouse model, the authors should indicate how many viruses should be added and when to measure in vivo fluorescence after pseudovirus infection.

Referee #3 (Comments on Novelty/Model System for Author):

1. Statistics could be improved (see specific comments). I would like to see an added control in the Co-IPs.
2. This is a very interesting and complete description of a new mechanism.
3. Currently, it's hard to estimate the actual impact the full mechanism, as described by the authors, has in COVID-19. Testing GLK-inhibition may reveal a potential new treatment route.
4. I don't see any issues here.

Referee #3 (Remarks for Author):

In their manuscript, Chuang et al. investigate the role of MAP4K3/GLK in a potential feedback cycle during SARS-CoV-2 infection. They find that
1. The SARS-CoV-2 spike protein induces the expression of GLK
2. GLK is linked to exosome numbers
3. These exosomes carry ACE2, the main SARS-CoV-2 entry receptor
4. The exosomes can transfer ACE2 to other cells
5. ACE2 protein levels are increased by GLK
6. This increase is mediated by direct interaction and phosphorylation of ACE2, in turn preventing ubiquitinylation and thus proteasomal degradation
7. These mechanisms can be observed in an ACE2-humanized mouse model system

From this they deduce that the S-protein sensitizes its host organism to infection by indirectly increasing the number of ACE2-carrying cells through the aforementioned mechanisms. I find the manuscript quite exciting and the individual mechanisms described to be plausible. One aspect I'm ot fully convinced of at this point is whether theproposed mechanism in its entirety plays a significant role in COVID-19: while it is possible that GLK induction was the cause of increased exosome numbers in COVID-19 patients (and mice), the spike in exosomes could simply have been due to tissue damage during viral infection. This does not at all invalidate the author's findings, as the increase of ACE2 protein levels would still be relevant and ACE2 ends up in exosomes with or without GLK in this scenario. However, it would limit the role of GLK in the process and make it appear as a less interesting target for potential interventions. Thus, it would be intersting to see how much of the exosome release is due to GLK expression, which could be checked in the mouse model by comparing amounts of exosomes in the presence/absence of GLK inhibition. In case the authors do not want to follow up on this, I would at least like to see this point discussed. Other than this, I only have comments that should be straightforward to address:

• The title of the manuscript uses the name MAP4K3, wheres the main text generally uses GLK. This should be harmonized.
• Figure 1C: it is rather unlikely, that the requirements for a t-test are satisfied here. The variability in OVID-19 patients is likely to be increased by factors such as within-group variability o disease course, time of sampling, etc. Thus, homogeneity of variance is rather unlikely. Furthermore, while it won't massively affect the results here, in principle a correction for multiple testing would be appropriate. The authors could consider switching to Dunn's test or a similar, non-parametric multi-comparison test (see below).
• Comparisons based on absolute cell counts alone are not very meaningful in (clinical) scRNA-Seq data, as samples may not have even cell numbers (e.g. with Chua et al., the controls have lower cell numbers). Also, the percentage of positive cells strongly depends on the sequencing depth which in turn may be affected by cell quality (especially percentage of reads in cells). Still, comparing percentage of positive cells or splitting the population and running tools such as scCoda would be more robust.
• Fig. 1 E/F are missing
• It's unclear what „simple" epithelial cells are supposed to be. Since ciliated cells are listed separately, would this refer to any epithelial cell type that's not ciliated (i.e. basal, goblet, club, ionocyte, FOXN4+, keratinocyte). In that case, is there a specific reason for this split? Basal, goblet, club, and ciliated cells belong to the same lineage, whereas the other cell types do not.
• Figure 3C: this roughly looks like there's distinct populations with respect to ACE2 levels within the COVID-19 cases, with patients 4, 7, and possibly 2 showing levels comparable to the healthy controls, at least when comparing to the total exosome number. Would the authors have any information regarding these patients, e.g. the severity of symptoms or time from onset of

symptoms?
• Figure 3D: the authors showed previously that GLK can increase the number of exosomes. If exosome production was mediated via some other mechanism (e.g. cell damage), the results could be explained by a lack of exosomes, unless similar amounts of exosomes were used per group.
• Figure 4B: could the authors demonstrate the absence of unspecific binding by having a +Myc, -FLAG control? Same goes for other Co-IPs.
• Figure 4G: could the auhors provide an estimate oft he half-life? The increase in baseline levels makes this a bit hard to determine visually
• Figure 4H: the addition of GLK does not seem to affect the ACE2 level here. Could the authors add some details about the timing (transfection to measurement) here? Was this shorter than for other experiments?
• Figure 6G: according to the labels in this panels, there is an ACE2-FLAG band in a sample where no FLAG-tagged protein should be present. Could the authors carefully check the labels here?
• Some antibody clones/catalog numbers appear to be missing (CD*)
• I did not get a hit fort he ACE2 primers listed using either UCSC Genome Browser's in silico PCR or Primer Blast (against genome or transcriptome). Are the sequences correct?

Reply to Reviewer #1:

The current manuscript titled "SARS-CoV-2 spike protein enhances MAP4K3-induced ACE2 stability in COVID-19" describes whether GLK is involved in COVID-19 pathogenesis by scRNA-seq and proteomics analyses using four COVID-19 patient cohorts from Taiwan. Interestingly, the results suggest that GLK stabilizes ACE2 proteins in epithelial cells, enhancing SARS-CoV-2 infection susceptibility of epithelial cells. More specifically, the authors found that both protein levels and mRNA levels of GLK were increased in pseudovirus-infected HCC827 lung epithelial cells, as well as GLK levels increasing with the spike protein. They also found increased levels of the exosome marker CD63 in serum exosomes suggesting an enhancement of exosome release in COVID-19 patients. In situ proximity ligation assays (PLA) with ACE2 showed PLA signals in cells co-transfected with ACE2 and Flag-GLK, suggested a direct interaction between ACE2 and GLK. They also utilized UBR4 overexpression which enhanced Lys48-linked ubiquitination of ACE2 leading to proteasomal degradation of ACE2. Specificity controls included use of ACE2 (K26R), ACE2 (K112R), and ACE2 (K114R) and ACE2 (K94R) mutation resistant to UBR4-induced ACE2 degradation. Overall, this is an interesting and novel manuscript, however there are few minor concerns that include:

Reply: We thank the encouraging and constructive comments from Reviewer #1.

Comment 1. *Have the authors performed any analysis on the exosomal contents? Are there any proteins related to the mitochondrial pathway? Any functional analysis of the exosomes on recipient cells?*

Reply: We have listed serum exosomal proteins identified from COVID-19 patients and healthy controls (Source Data). The Cellular Component Ontology analysis shows that COVID-19 patient-enriched exosomal proteins mainly belong to membrane proteins, lysosomal proteins, and mitochondria proteins, while healthy control-enriched exosomal proteins mainly belong to cytosolic proteins and nuclear proteins (Appendix Fig S5). Moreover, KEGG pathway analysis shows that COVID-19 patient-enriched exosomal proteins may be involved in multiple metabolic pathways (Appendix Fig S6; page 7, line 16 to page 8, line 4). In addition, our results showed that GLK-induced ACE2-containing exosomes facilitated SARS-CoV-2 infection (Fig 3F, 7G, and 7H).

Comment 2. *Data in Figure 7 is very interesting, but somewhat overwhelming! Can the authors break up this figure into two and explain the results better in the result and discussion sections with more depth?*

Reply: Due to the limitation of the Figure number, we could not break up Figure 7. Nevertheless, we have re-organized Figure 7 (page 17, line 16 to page 18, line 9) and added subtitle to the result section (page 18, lines 10-11).

Reply to Reviewer #2:

In this manuscript, Chuang et al found that SARS-CoV-2 infection, particularly the spike protein, can induce the GLK expression. In addition, GLK can induce release of exosome with ACE2 and stabilize ACE2 by reducing ubiquitination, thus promoting the SARS-CoV-2 infection. It is an interesting and novel pathway of SARS-CoV-2 pathogenesis, which will attract wide attentions from virologist. This pathway can also serve as a therapeutic target for COVID-19 treatment.

This manuscript is well organized. The references are correctly cited. The methods are well described. However, some modifications should be made to further improve the solidarity of the conclusion.

Reply: We appreciate the encouraging and constructive comments from Reviewer #2.

Comment 1. *In figure 1C and D, and EV2, the figure legend indicates percentage while the figures show cell counts. The increase in GLK+ cell count cannot support that GLK expression was induced by SARS-CoV-2 infection since the total cell count in BLAF in SARS-CoV-2 patients are increased. So the percentage should be used here. Besides, the statistical analysis should use ANOVA instead of t-test since there are more than 2 groups. Same problem is also in figure 4I.*

Reply: We appreciate the constructive suggestion from Reviewer #2. Per Reviewer's comment, we have changed the data from cell count to the percentage (Figure 1C and 1D) accordingly. Moreover, we have performed the statistical analysis of Figure 1C, 1D, 3F, 4I, and 4J using *ANOVA* instead of *t-test* analysis accordingly (page 35, line 4; page 38, line 4; page 40, line 4).

Comment 2. *To test whether SARS-CoV-2 infection results in GLK overexpression in lung epithelial cells, the author used SARS-CoV-2 pseudovirus. A control pseudovirus with other viral surface protein should be added to see whether this GLK overexpression is specifically induced by SARS-CoV-2.*

Reply: Per Reviewer's comment, we have added vesicular stomatitis virus-G (VSV-G) pseudotyped lentivirus as a control pseudovirus and found that GLK levels were induced by SARS-CoV-2 but not VSV-G (Appendix Fig S4; page 5, lines 18-19).

Comment 3. *Similarly, in figure 3F, another pseudovirus should be used here to confirm whether the enhanced viral infection results from increased ACE2 level or other non-specific mechanism, such as enhanced endocytosis. Same problem is also in figure 4I.*

Reply: Per Reviewer's comment, we have treated GLK-overexpressing HCC827 epithelial cells with SARS-CoV-2 and VSV-G pseudovirus and found that "GLK overexpression did not enhance infection of VSV-G pseudovirus (Fig 4J)" (page 12, lines 12-13).

Comment 4. *In figure 4D, why the lower bands of ACE2 contains 3 bands.*

Reply: We have replaced Fig 4D with a clear figure.

Comment 5. *The quality of Figure 5A is poor.*

Reply: Per Reviewer's comment, we have replaced Fig 5A with a high-quality figure.

Minors:

Comment 1. *In page 5, the author mentioned figure 1E and F, but no figure 1E and F in the figure section?*

Reply: We have provided the data for Fig 1E and 1F, which were mistakenly placed in Expanded View Figures.

Comment 2. *In methods section -- SARS-CoV-2 pseudovirus infection mouse model, the authors should indicate how many viruses should be added and when to measure in vivo fluorescence after pseudovirus infection.*

Reply: Per Reviewer's comment, we have added the virus titer and measurement time point to the SARS-CoV-2 pseudovirus infection mouse model section in Materials and Methods (page 25, lines 7 and 10).

<u>Reply to Reviewer #3:</u>

Comments on Novelty/Model System for Author

1. Statistics could be improved (see specific comments). I would like to see an added control in the Co-IPs.

2. This is a very interesting and complete description of a new mechanism.

3. Currently, it's hard to estimate the actual impact the full mechanism, as described by the authors, has in COVID-19. Testing GLK-inhibition may reveal a potential new treatment route.

4. I don't see any issues here.

Reply: Per Reviewer's comment, we have improved statistical analyses (see below) and have added a control in the co-immunoprecipitation experiments (Fig 4B and 4H). Furthermore, we have added data of GLK inhibition in Fig EV5 (page 19, lines 3-15).

Remarks for Author

In their manuscript, Chuang et al. investigate the role of MAP4K3/GLK in a potential feedback cycle during SARS-CoV-2 infection. They find that

1. The SARS-CoV-2 spike protein induces the expression of GLK

2. GLK is linked to exosome numbers

3. These exosomes carry ACE2, the main SARS-CoV-2 entry receptor

4. The exosomes can transfer ACE2 to other cells
5. ACE2 protein levels are increased by GLK
6. This increase is mediated by direct interaction and phosphorylation of ACE2, in turn preventing ubiquitinylation and thus proteasomal degradation
7. These mechanisms can be observed in an ACE2-humanized mouse model system
From this they deduce that the S-protein sensitizes its host organism to infection by indirectly increasing the number of ACE2-carrying cells through the aforementioned mechanisms. I find the manuscript quite exciting and the individual mechanisms described to be plausible. One aspect I'm not fully convinced of at this point is whether the proposed mechanism in its entirety plays a significant role in COVID-19: while it is possible that GLK induction was the cause of increased exosome numbers in COVID-19 patients (and mice), the spike in exosomes could simply have been due to tissue damage during viral infection. This does not at all invalidate the author's findings, as the increase of ACE2 protein levels would still be relevant and ACE2 ends up in exosomes with or without GLK in this scenario. However, it would limit the role of GLK in the process and make it appear as a less interesting target for potential interventions. Thus, it would be interesting to see how much of the exosome release is due to GLK expression, which could be checked in the mouse model by comparing amounts of exosomes in the presence/absence of GLK inhibition. In case the authors do not want to follow up on this, I would at least like to see this point discussed.

Reply: We thank the encouraging and constructive comments from Reviewer #3. Per Reviewer's comment #7, we have added the potential role of GLK in the enhancement of exosome release to the discussion section (page 9, lines 17-19; page 21, lines 3-5).

Other than this, I only have comments that should be straightforward to address:

Comment 1. *The title of the manuscript uses the name MAP4K3, whereas the main text generally uses GLK. This should be harmonized.*

Reply: Per Reviewer's comment, we have added "GLK" to the title (page 1, line 1).

Comment 2. *Figure 1C: it is rather unlikely, that the requirements for a t-test are satisfied here. The variability in COVID-19 patients is likely to be increased by factors such as within-group variability of disease course, time of sampling, etc. Thus, homogeneity of variance is rather unlikely. Furthermore, while it won't massively affect the results here, in principle a correction for multiple testing would be appropriate. The authors could consider switching to Dunn's test or a similar, non-parametric multi-comparison test (see below).*

Reply: Per Reviewer #3 (and also Reviewer #2)'s comments, we have performed the statistical analysis of Fig 1C, 1D using *ANOVA* instead of *t-test* analysis (page 35, line 4).

Comment 3. *Comparisons based on absolute cell counts alone are not very meaningful in (clinical) scRNA-Seq data, as samples may not have even cell numbers (e.g. with Chua et al., the controls have lower cell numbers). Also, the percentage of positive cells strongly depends on the sequencing depth which in turn may be affected by cell quality (especially percentage of reads in cells). Still, comparing percentage of positive cells or splitting the population and running tools such as scCoda would be more robust.*

Reply: Per Reviewer's comment, we have changed the data from cell count to the percentage (Figure 1C and 1D) accordingly (page 5, lines 2-5). Moreover, we have provided GLK levels in epithelial subgroups of individuals from Cohort #1 and Cohort #2; these results were statistically analyzed by *Kruskal-Wallis* analysis (Fig 1E, 1F and Appendix Fig S3C, S3D; page 5, lines 5-7 and 13-14).

Comment 4. *Fig. 1 E/F are missing.*

Reply: We have provided the data for Fig 1E and 1F, which were mistakenly placed in Expanded View Figures.

Comment 5. *It's unclear what „simple" epithelial cells are supposed to be. Since ciliated cells are listed separately, would this refer to any epithelial cell type that's not ciliated (i.e. basal, goblet, club, ionocyte, FOXN4+, keratinocyte). In that case, is there a specific reason for this split? Basal, goblet, club, and ciliated cells belong to the same lineage, whereas the other cell types do not.*

Reply: Per Reviewer's comment, we have changed "simple epithelial cells" to "KRT18$^+$ epithelial cells" (page 5, line 2).

Comment 6. *Figure 3C: this roughly looks like there's distinct populations with respect to ACE2 levels within the COVID-19 cases, with patients 4, 7, and possibly 2 showing levels comparable to the healthy controls, at least when comparing to the total exosome number. Would the authors have any information regarding these patients, e.g. the severity of symptoms or time from onset of symptoms?*

Reply: Per Reviewer's comment, we have added the day from onset of COVID-19 patients and the treatment of oxygen therapy to Fig 3C (page 37, lines 4-5).

Comment 7. *Figure 3D: the authors showed previously that GLK can increase the number of exosomes. If exosome production was mediated via some other mechanism (e.g. cell damage), the results could be explained by a lack of exosomes, unless similar amounts of exosomes were used per group.*

Reply: For Fig 3D, we have previously demonstrated that GLK overexpression in epithelial

cells does not cause cell damage/death (Chuang *et al*., 2019a), and there is no SARS-CoV-2 infection in Fig 3D. Nevertheless, we have added the sentence "we cannot rule out the possibility that SARS-CoV-2 infection may induce epithelial cell damage in COVID-19 patients, resulting in enhancement of exosome release" to the discussion section (page 21, lines 3-5).

Comment 8. *Figure 4B: could the authors demonstrate the absence of unspecific binding by having a +Myc, -FLAG control? Same goes for other Co-IPs.*

Reply: Per Reviewer's comment, we have added a +Myc, -FLAG control in Fig 4B. We have also added a negative control in Fig 4H.

Comment 9. *Figure 4G: could the authors provide an estimate of the half-life? The increase in baseline levels makes this a bit hard to determine visually.*

Reply: Per Reviewer's comment, we have added the estimated half-life of ACE2 protein to the sentence that "GLK overexpression prolonged ACE2 protein half-life (estimated half-life: 39.1 h to 157.5 h) in HEK293T cells" (page 12, line 5).

Comment 10. *Figure 4H: the addition of GLK does not seem to affect the ACE2 level here. Could the authors add some details about the timing (transfection to measurement) here? Was this shorter than for other experiments?*

Reply: The ratio of GLK to ACE2 was 1 in the original Fig 4H. In this revised manuscript, we have re-performed the experiment for Fig 4H under a condition that the ratio of GLK to ACE2 was 2.

Comment 11. *Figure 6G: according to the labels in this panels, there is an ACE2-FLAG band in a sample where no FLAG-tagged protein should be present. Could the authors carefully check the labels here?*

Reply: We have carefully checked the labels of Fig 6G and other Figures in this manuscript; we have corrected the typo error in Fig 6G.

Comment 12. *Some antibody clones/catalog numbers appear to be missing (CD*)*

Reply: We have added the clone number of anti-CD9 antibody to the Materials and Methods section (page 26, lines 19-20).

Comment 13. *I did not get a hit for the ACE2 primers listed using either UCSC Genome Browser's in silico PCR or Primer Blast (against genome or transcriptome). Are the sequences correct?*

Reply: We would like to clarify that in order to optimize the translation of human ACE2 mRNA

in mice, the sequences of hACE2 transgenic mice were modified using murine-optimized codons; the codon-optimized ACE2 cDNA sequences have been provided in Source Data 2 (page 24, line 21 and page 25, line 2).

4th Jul 2022

Dear Prof. Tan,

Thank you for the submission of your revised manuscript to EMBO Molecular Medicine. I am pleased to inform you that we will be able to accept your manuscript pending the following final amendments:

1) Please address the referee's comment by performing the appropriate statistical test.
2) In the main manuscript file, please do the following:
- In M&M, provide the antibody dilutions that were used for each antibody.
- In M&M, statistical paragraph should reflect all information that you have filled in the Authors Checklist, especially regarding randomization, blinding, replication.
- In M&M, include a statement that informed consent was obtained from all human subjects and that, in addition to the WMA Declaration of Helsinki , the experiments conformed to the principles set out in the Department of Health and Human Services Belmont Report.
3) Authors Checklist: Please select "Yes" or "Not Applicable" in the column "Information included in the manuscript".
4) For more information: Please remove corresponding author's e-mail address. This space should be used to list relevant web links for further consultation by our readers. Could you identify some relevant ones and provide such information as well? Some examples are patient associations, relevant databases, OMIM/proteins/genes links, author's websites, etc...
5) Press release: Please inform us as soon as possible and latest at the time of submission of the revised manuscript if you plan a press release for your article so that our publisher could coordinate publication accordingly.
6) Please be aware that we use a unique publishing workflow for COVID-19 papers: a non-typeset PDF of the accepted manuscript is published as "Just Accepted" on our website. With respect to a possible press release, we have the option to not post the "Just Accepted" version if you prefer to wait with the press release for the typeset version. Please let us know whether you agree to publication of a "Just accepted" version or you prefer to wait for the typeset version.
7) As part of the EMBO Publications transparent editorial process initiative (see our Editorial at http://embomolmed.embopress.org/content/2/9/329), EMBO Molecular Medicine will publish online a Review Process File (RPF) to accompany accepted manuscripts. This file will be published in conjunction with your paper and will include the anonymous referee reports, your point-by-point response and all pertinent correspondence relating to the manuscript. Let us know whether you agree with the publication of the RPF and as here, if you want to remove or not any figures from it prior to publication. Please note that the Authors checklist will be published at the end of the RPF.
8) Please provide a point-by-point letter INCLUDING my comments as well as the reviewer's reports and your detailed responses (as Word file).

I look forward to reading a new revised version of your manuscript as soon as possible.

Yours sincerely,

Zeljko Durdevic

Zeljko Durdevic
Editor
EMBO Molecular Medicine

***** Reviewer's comments *****

Referee #3 (Comments on Novelty/Model System for Author):

The manuscript puts forward novel and exciting ideas, shows the required rigor, and highlights potential future avenues for undersanding and treating COVID-19 better. The medical impact is not immediate, as the findings are not actionable at the moment, but may very well be in the future.

Referee #3 (Remarks for Author):

The authors have addressed my concerns comprehensively and alleviated reservations I had about some results. As requested by the editor, I also checked the response to reviewer 2 and am confident that the concerns were adequately addressed. I recommend the manuscript for publication, but have one small request: Reviewer 2 and I commented on the statistics for Figures 1C and D and 4I. Switching to ANOVA is an improvement, but could the authors add a post-hoc test (Dunn's or Dunnett's test) on top to show differences between groups? The differences are clear, so this will not require re-review.

Reply to Reviewer #3:

Comments on Novelty/Model System for Author

The manuscript puts forward novel and exciting ideas, shows the required rigor, and highlights potential future avenues for undersanding and treating COVID-19 better. The medical impact is not immediate, as the findings are not actionable at the moment, but may very well be in the future.

Reply: We thank the encouraging and constructive comments from Reviewer #3.

Remarks for Author

The authors have addressed my concerns comprehensively and alleviated reservations I had about some results. As requested by the editor, I also checked the response to reviewer 2 and am confident that the concerns were adequately addressed. I recommend the manuscript for publication, but have one small request: Reviewer 2 and I commented on the statistics for Figures 1C and D and 4I. Switching to ANOVA is an improvement, but could the authors add a post-hoc test (Dunn's or Dunnett's test) on top to show differences between groups? The differences are clear, so this will not require re-review.

Reply: Per Reviewer's comment, we have added a *post-hoc tes*t (*Dunnett's test*) to Figure 1C, 1D, and 4I.

We are pleased to inform you that your manuscript is accepted for publication and is now being sent to our publisher to be included in the next available issue of EMBO Molecular Medicine.

EMBO Press Author Checklist

Corresponding Author Name: Doryen Bubeck and Inacio Moraga	
Journal Submitted to: EMBO Reports	
Manuscript Number: EMBOR-2022-55450-T	

USEFUL LINKS FOR COMPLETING THIS FORM
The EMBO Journal - Author Guidelines
EMBO Reports - Author Guidelines
Molecular Systems Biology - Author Guidelines
EMBO Molecular Medicine - Author Guidelines

Reporting Checklist for Life Science Articles (updated January 2022)
This checklist is adapted from Materials Design Analysis Reporting (MDAR) Checklist for Authors. MDAR establishes a minimum set of requirements in transparent reporting in the life sciences (see Statement of Task: 10.31222/osf.io/9sm4x). Please follow the journal's guidelines in preparing your manuscript.
Please note that a copy of this checklist will be published alongside your article.

Abridged guidelines for figures
1. Data
The data shown in figures should satisfy the following conditions:
- the data were obtained and processed according to the field's best practice and are presented to reflect the results of the experiments in an accurate and unbiased manner.
- ideally, figure panels should include only measurements that are directly comparable to each other and obtained with the same assay.
- plots include clearly labeled error bars for independent experiments and sample sizes. Unless justified, error bars should not be shown for technical replicates.
- if n<5, the individual data points from each experiment should be plotted. Any statistical test employed should be justified.
- Source Data should be included to report the data underlying figures according to the guidelines set out in the authorship guidelines on Data Presentation.

2. Captions
Each figure caption should contain the following information, for each panel where they are relevant:
- a specification of the experimental system investigated (eg cell line, species name).
- the assay(s) and method(s) used to carry out the reported observations and measurements.
- an explicit mention of the biological and chemical entity(ies) that are being measured.
- an explicit mention of the biological and chemical entity(ies) that are altered/varied/perturbed in a controlled manner.
- the exact sample size (n) for each experimental group/condition, given as a number, not a range;
- a description of the sample collection allowing the reader to understand whether the samples represent technical or biological replicates (including how many animals, litters, cultures, etc.).
- a statement of how many times the experiment shown was independently replicated in the laboratory.
- definitions of statistical methods and measures:
  - common tests, such as t-test (please specify whether paired vs. unpaired), simple χ2 tests, Wilcoxon and Mann-Whitney tests, can be unambiguously identified by name only, but more complex techniques should be described in the methods section;
  - are tests one-sided or two-sided?
  - are there adjustments for multiple comparisons?
  - exact statistical test results, e.g., P values = x but not P values < x;
  - definition of 'center values' as median or average;
  - definition of error bars as s.d. or s.e.m.

Please complete ALL of the questions below.
Select "Not Applicable" only when the requested information is not relevant for your study.

Materials

Newly Created Materials	Information included in the manuscript?	In which section is the information available? (Reagents and Tools Table, Materials and Methods, Figures, Data Availability Section)
New materials and reagents need to be available; do any restrictions apply?	Not Applicable	There are no restrictions on new material generated and are available upon request.

Antibodies	Information included in the manuscript?	In which section is the information available? (Reagents and Tools Table, Materials and Methods, Figures, Data Availability Section)
For **antibodies** provide the following information:<br>- Commercial antibodies: RRID (if possible) or supplier name, catalogue number and or/clone number<br>- Non-commercial: RRID or citation	Not Applicable	

DNA and RNA sequences	Information included in the manuscript?	In which section is the information available? (Reagents and Tools Table, Materials and Methods, Figures, Data Availability Section)
**Short novel DNA or RNA including primers, probes**: provide the sequences.	Not Applicable	

Cell materials	Information included in the manuscript?	In which section is the information available? (Reagents and Tools Table, Materials and Methods, Figures, Data Availability Section)
**Cell lines:** Provide species information, strain. Provide accession number in repository **OR** supplier name, catalog number, clone number, and/**OR** RRID.	Yes	Materials and Methods
**Primary cultures:** Provide species, strain, sex of origin, genetic modification status.	Yes	Materials and Methods
Report if the cell lines were recently **authenticated** (e.g., by STR profiling) and tested for mycoplasma contamination.	Not Applicable	

Experimental animals	Information included in the manuscript?	In which section is the information available? (Reagents and Tools Table, Materials and Methods, Figures, Data Availability Section)
**Laboratory animals or Model organisms:** Provide species, strain, sex, age, genetic modification status. Provide accession number in repository **OR** supplier name, catalog number, clone number, **OR** RRID.	Not Applicable	
**Animal observed in or captured from the field:** Provide species, sex, and age where possible.	Not Applicable	
Please detail **housing and husbandry conditions**.	Not Applicable	

Plants and microbes	Information included in the manuscript?	In which section is the information available? (Reagents and Tools Table, Materials and Methods, Figures, Data Availability Section)
**Plants:** provide species and strain, ecotype and cultivar where relevant, unique accession number if available, and source (including location for collected wild specimens).	Not Applicable	
**Microbes:** provide species and strain, unique accession number if available, and source.	Not Applicable	

Human research participants	Information included in the manuscript?	In which section is the information available? (Reagents and Tools Table, Materials and Methods, Figures, Data Availability Section)
If collected and within the bounds of privacy constraints report on age, sex and gender or ethnicity for all study participants.	Not Applicable	

Core facilities	Information included in the manuscript?	In which section is the information available? (Reagents and Tools Table, Materials and Methods, Figures, Data Availability Section)
If your work benefited from core facilities, was their service mentioned in the acknowledgments section?	Yes	Acknowledgements

Design

Study protocol	Information included in the manuscript?	In which section is the information available? (Reagents and Tools Table, Materials and Methods, Figures, Data Availability Section)
If study protocol has been **pre-registered, provide DOI in the manuscript**. For clinical trials, provide the trial registration number **OR** cite DOI.	Not Applicable	
Report the **clinical trial registration number** (at ClinicalTrials.gov or equivalent), where applicable.	Not Applicable	

Laboratory protocol	Information included in the manuscript?	In which section is the information available? (Reagents and Tools Table, Materials and Methods, Figures, Data Availability Section)
Provide DOI OR other citation details if **external detailed step-by-step protocols** are available.	Not Applicable	

Experimental study design and statistics	Information included in the manuscript?	In which section is the information available? (Reagents and Tools Table, Materials and Methods, Figures, Data Availability Section)
Include a statement about **sample size** estimate even if no statistical methods were used.	Yes	Figure legends
Were any steps taken to minimize the effects of subjective bias when allocating animals/samples to treatment (e.g. **randomization procedure**)? If yes, have they been described?	Not Applicable	
Include a statement about **blinding** even if no blinding was done.	Not Applicable	
Describe **inclusion/exclusion criteria** if samples or animals were excluded from the analysis. Were the criteria pre-established?<br><br>If sample or data points were omitted from analysis, report if this was due to attrition or intentional exclusion and provide justification.	Yes	Information describing how particles were selected at different points of the image processing workflow are included in Materials and Methods.
For every figure, are **statistical tests** justified as appropriate? Do the data meet the assumptions of the tests (e.g., normal distribution)? Describe any methods used to assess it. Is there an estimate of variation within each group of data? Is the variance similar between the groups that are being statistically compared?	Not Applicable	

Sample definition and in-laboratory replication	Information included in the manuscript?	In which section is the information available? (Reagents and Tools Table, Materials and Methods, Figures, Data Availability Section)
In the figure legends: state number of times the experiment was **replicated** in laboratory.	Yes	Figure legend
In the figure legends: define whether data describe **technical or biological replicates**.	Yes	Figure legend

Ethics

Ethics	Information included in the manuscript?	In which section is the information available? (Reagents and Tools Table, Materials and Methods, Figures, Data Availability Section)
Studies involving **human participants**: State details of **authority granting ethics approval** (IRB or equivalent committee(s), provide reference number for approval.	Not Applicable	
Studies involving **human participants**: Include a statement confirming that **informed consent** was obtained from all subjects and that the experiments conformed to the principles set out in the WMA Declaration of Helsinki and the Department of Health and Human Services Belmont Report.	Not Applicable	
Studies involving **human participants:** For publication of **patient photos**, include a statement confirming that consent to publish was obtained.	Not Applicable	
Studies involving experimental **animals**: State details of **authority granting ethics approval** (IRB or equivalent committee(s), provide reference number for approval. Include a statement of compliance with ethical regulations.	Yes	Matherials and Methods
Studies involving **specimen and field samples:** State if relevant **permits** obtained, provide details of authority approving study; if none were required, explain why.	Not Applicable	

Dual Use Research of Concern (DURC)	Information included in the manuscript?	In which section is the information available? (Reagents and Tools Table, Materials and Methods, Figures, Data Availability Section)
Could your study fall under dual use research restrictions? Please check biosecurity documents and list of **select agents and toxins** (CDC): https://www.selectagents.gov/sat/list.htm	Not Applicable	
If you used a select agent, is the security level of the lab appropriate and reported in the manuscript?	Not Applicable	
If a study is subject to dual use research of concern regulations, is the name of the **authority granting approval and reference number** for the regulatory approval provided in the manuscript?	Not Applicable	

Reporting

The MDAR framework recommends adoption of discipline-specific guidelines, established and endorsed through community initiatives. Journals have their own policy about requiring specific guidelines and recommendations to complement MDAR.

Adherence to community standards	Information included in the manuscript?	In which section is the information available? (Reagents and Tools Table, Materials and Methods, Figures, Data Availability Section)
State if relevant guidelines or checklists (e.g., **ICMJE, MIBBI, ARRIVE, PRISMA**) have been followed or provided.	Not Applicable	
For **tumor marker prognostic studies,** we recommend that you follow the **REMARK** reporting guidelines (see link list at top right). See author guidelines, under 'Reporting Guidelines'. Please confirm you have followed these guidelines.	Not Applicable	
For **phase II and III randomized controlled trials**, please refer to the **CONSORT** flow diagram (see link list at top right) and submit the CONSORT checklist (see link list at top right) with your submission. See author guidelines, under 'Reporting Guidelines'. Please confirm you have submitted this list.	Not Applicable	

Data Availability

Data availability	Information included in the manuscript?	In which section is the information available? (Reagents and Tools Table, Materials and Methods, Figures, Data Availability Section)
Have **primary datasets** been deposited according to the journal's guidelines (see 'Data Deposition' section) and the respective accession numbers provided in the Data Availability Section?	Yes	Data Availability Section.
Were **human clinical and genomic datasets** deposited in a public access-controlled repository in accordance to ethical obligations to the patients and to the applicable consent agreement?	Not Applicable	
Are **computational models** that are central and integral to a study available without restrictions in a machine-readable form? Were the relevant accession numbers or links provided?	Not Applicable	
If publicly available data were reused, provide the respective **data citations in the reference list**.	Yes	Reference list in the main text
